# Supplementary material for: Identifying common health indicators from paediatric core outcome sets: a systematic review with narrative synthesis using the WHO International Classification of Functioning, Health and Disability
Source: BMJ Paediatr Open. 2022 Oct 28;6(1):e001537. doi: 10.1136/bmjpo-2022-001537 (PMC9621176; doi:10.1136/bmjpo-2022-001537)
Supplement: Author's manuscript [file bmjpo-2022-001537.draft_revisions.pdf]

BMJ Paediatrics Open

BMJ Paediatrics Open is committed to open peer review. As part of this commitment we make the peer review history of every article we publish publicly available.

When an article is published we post the peer reviewers' comments and the authors' responses online. We also post the versions of the paper that were used during peer review. These are the versions that the peer review comments apply to.

The versions of the paper that follow are the versions that were submitted during the peer review process. They are not the versions of record or the final published versions. They should not be cited or distributed as the published version of this manuscript.

BMJ Paediatrics Open is an open access journal and the full, final, typeset and author-corrected version of record of the manuscript is available on our site with no access controls, subscription charges or pay-per-view fees (http://bmjpaedsopen.bmj.com).

If you have any questions on BMJ Paediatrics Open's open peer review process please email info.bmjpo@bmj.com

BMJ Paediatrics Open

Identifying common health indicators from paediatric core outcome sets: a scoping review and mapping on the WHO International Classification of Functioning, Health and Disability

Journal:	*BMJ Paediatrics Open*
Manuscript ID	bmjpo-2022-001537
Article Type:	Review
Date Submitted by the Author:	05-May-2022
Complete List of Authors:	Harbottle, Victoria; Great North Children's Hospital, Rehabilitation Department; Newcastle University, Population Health Sciences Institute<br>Arnott, Bronia; Newcastle University, Population Health Sciences Institute<br>Gale, Chris; Imperial College London, Academic Neonatal Medicine<br>Rowen, Elizabeth; Great North Children's Hospital, Rehabilitation Department<br>Kolehmainen, Niina; Newcastle University, Population Health Sciences Institute; Newcastle Upon Tyne Hospitals NHS Foundation Trust,
Keywords:	Data Collection, Adolescent Health, Epidemiology

SCHOLARONE™
Manuscripts

I, the Submitting Author has the right to grant and does grant on behalf of all authors of the Work (as defined in the below author licence), an exclusive licence and/or a non-exclusive licence for contributions from authors who are: i) UK Crown employees; ii) where BMJ has agreed a CC-BY licence shall apply, and/or iii) in accordance with the terms applicable for US Federal Government officers or employees acting as part of their official duties; on a worldwide, perpetual, irrevocable, royalty-free basis to BMJ Publishing Group Ltd ("BMJ") its licensees and where the relevant Journal is co-owned by BMJ to the co-owners of the Journal, to publish the Work in this journal and any other BMJ products and to exploit all rights, as set out in our licence.

The Submitting Author accepts and understands that any supply made under these terms is made by BMJ to the Submitting Author unless you are acting as an employee on behalf of your employer or a postgraduate student of an affiliated institution which is paying any applicable article publishing charge ("APC") for Open Access articles. Where the Submitting Author wishes to make the Work available on an Open Access basis (and intends to pay the relevant APC), the terms of reuse of such Open Access shall be governed by a Creative Commons licence – details of these licences and which Creative Commons licence will apply to this Work are set out in our licence referred to above.

Other than as permitted in any relevant BMJ Author's Self Archiving Policies, I confirm this Work has not been accepted for publication elsewhere, is not being considered for publication elsewhere and does not duplicate material already published. I confirm all authors consent to publication of this Work and authorise the granting of this licence.

Confidential: For Review Only

Identifying common health indicators from paediatric core outcome sets: a scoping review and mapping on the WHO International Classification of Functioning, Health and Disability

Authors: Victoria Harbottle, Bronia Arnott, Chris Gale, Elizabeth Rowen, Niina Kolehmainen

Victoria Harbottle (Corresponding author)

Great North Children's Hospital, Royal Victoria Infirmary, Queen Victoria Road, Newcastle upon Tyne, NE1 4LP

Population Health Sciences Institute, Newcastle University, Richardson Road, Newcastle University, Newcastle upon Tyne, NE2 4AX, United Kingdom

victoria.harbottle@ncl.ac.uk

Bronia Arnott

Population Health Sciences Institute, Newcastle University, Newcastle upon Tyne, United Kingdom

Chris Gale

Neonatal Medicine, School of Public Health, Faculty of Medicine, Imperial College London, London, United Kingdom

Elizabeth Rowen

Great North Children's Hospital, Newcastle upon Tyne, United Kingdom

Niina Kolehmainen

Population Health Sciences Institute, Newcastle University, Newcastle upon Tyne, United Kingdom

Confidential: For Review Only

Great North Children's Hospital, Newcastle upon Tyne NHS Hospitals Trust, Newcastle upon Tyne, United Kingdom

Word count (excluding title page, abstract, references, figures and tables): 2968

Confidential: For Review Only

ABSTRACT

Background

Indicators of child health have the potential to inform societal conversations, decision making, and prioritisation. Paediatric core outcome sets are an increasingly common way of identifying a minimum set of outcomes for trials within clinical groups. Exploring commonality across existing sets may give insight into universally important and inclusive child health indicators.

Methods

A search of the Core Outcome Measures in Effectiveness Trial register from 2008 to 2022 was carried out. Eligible articles were those reporting on core outcome sets focused on children and young people aged 0-18 years old. The International Classification of Functioning, Disability and Health (ICF) was used as a framework to categorise extracted outcomes. Information about the involvement of children, young people and their families in the development of sets was also extracted.

Results

206 articles were identified, of which 36 were included. 441 unique outcomes were extracted, mapping to 22 outcome clusters present across multiple sets. Medical diagnostic outcomes were the biggest cluster, followed by pain, communication and social interaction, mobility, self-care and school. Children and young people's views were underrepresented

across core outcome sets, with only 36% of reviewed studies including them at any stage of development.

Conclusions

Existing paediatric core outcome sets show overlap in key outcomes, suggesting the potential for generic child health measurement frameworks. It is unclear whether existing sets best reflect health dimensions important to children and young people, and there is a need for better child and young person involvement in health indicator development to address this.

What is already known on this topic?

- There are aspects of children and young people's health that are not routinely, universally measured
- This lack of data about children and young people's health can hinder health policy, care and research
- Core outcome sets are a way of providing guidance on what to measure, but often relate to specific clinical populations only

What this study hopes to add?

- This study identifies common, universally shared themes across core outcome sets
- These themes help identify key universal indicators of child health that should be measured across populations
- The study further identifies gaps and areas for improvement in the involvement of children and their families in identifying key health indicators

Confidential: For Review Only

INTRODUCTION

Measure what is measurable, and make measurable what is not. (Isaac Newton)

How society measures characteristics of health is important because the act of measuring is an act of making an aspect of health visible—in societal discussion, decision making, and resource allocation. Measurable health characteristics are often referred to as indicators, and how they are defined for measurement shapes the information that is collected and available for decisions. Measuring health through routine and other large-scale data sets is increasingly common, and the use of the resulting data in societal conversations, decision making, and prioritisation likely has major consequences for people's lives.

Many important aspects of child health are not yet routinely measured. While some health indicators such as births, deaths and vaccinations are long-established, many others lack robust data. The Covid-19 pandemic provides an illustrative example, where the absence of data about children's physical and mental health, and development has hindered analyses of the impacts of the pandemic on children and, arguably, limited families', decision-makers', and professionals' ability to articulate their concerns. This, in turn, has hindered effective decision-making about important policy decisions such as opening of schools. The need to re-think the measurement of child health is gaining policy traction internationally[1-3] and the choice over what indicators to measure is now paramount.[4]

Measuring child health is hindered by lack of agreement about the important, universally applicable and clinically meaningful indicators of child health and well-being. The past decade has seen a positive, increased focus on common research outcomes relevant across professional disciplines, illustrated for example in the development of core outcome sets. Core outcome sets provide an agreed, standardised minimum set of outcomes to be

Confidential: For Review Only

reported for a specific clinical group in clinical research using routine patient data and increasingly in clinical practice.[5] However, although patient involvement in core outcome set development has been recognised important,[6] the sets continue to be fundamentally organised around clinical and diagnostic groups to service healthcare and trials. They do not currently provide an off-the-shelf set for universally important indicators of child health that could be applied across diagnostic categories, and across healthy and clinical populations.

We believe there is a need for a generic, universal, inclusive set of core child health and well-being indicators, and we think such a set should: (i) take the child's perspective (as opposed to e.g. medical or educational); (ii) adopt a life-course view, i.e. consider future health and well-being prospects as well as present health; (iii) be practically oriented, with real potential to inform routine data collection and societal discourse, policy, and interventions; and (iv) be inclusive of diverse health and developmental trajectories.

As the first step, in 2017-2020, we engaged with young people, families and decision-makers to discuss broad ideas about health indicators that might matter to them. Informed by those discussions, the present study sought to identify common themes in existing paediatric core outcome sets, as a way to contribute to the wider efforts to progress towards a generic, universal, inclusive set of core child health and well-being indicators. The present paper reports on two specific objectives: (i) to identify common indicators of health included in published paediatric core outcome sets for children and youth (0-18yrs); and (ii) to explore how children, young people and their families' perspectives informed the selection of these outcomes.

METHODS

This study used established review methods[7,8] to identify and select papers reporting on published paediatric core outcome sets. A protocol was agreed by the review team, with objectives, selection criteria and data extraction techniques agreed in advance. The results

Confidential: For Review Only

are reported in accordance with the Preferred Reporting Items for Systematic Reviews and Meta Analyses extension for Scoping Reviews (PRISMA-ScR) statement.[9] The review objectives fell outside the scope of the International Prospective Register of Systematic Reviews (PROSPERO).[10] We used the WHO International Classification of Functioning, Disability and Health (ICF),[11] an existing consensus framework of health, to provide multidisciplinary terminology for discussing health indicators across body functions and structures, activity, and participation.

Search

Paediatric core outcome sets were identified from the Core Outcome Measures in Effectiveness Trial (COMET) register.[12] The register is the internationally recognised database of ongoing and completed studies relating to core outcome set development. It is updated by systematic review annually, and by authors registering their sets on an ad hoc basis. The last review was published 01/2021 and includes studies up to and including 12/2018.[13] To identify paediatric sets, we systematically searched the COMET register[12] from 01/2008 to 03/2022 using the terms indicated in table 1. The search was updated to incorporate the COMET database review up to and including 12/2019.[14]

Selection

Articles were eligible for inclusion in this review if: (i) they reported on a disease or diagnostic-group specific core outcome sets developed using consensus methods (defined as reporting a clear criteria for determining outcome inclusion/exclusion, the number of people in each stakeholder group at each stage, and all outcome scoring[6]); (ii) focused on children and young people (0-18yrs) as the health beneficiaries; AND (iii) was published after 2008 (a previous systematic review[15] identifies work prior to this date). Systematic reviews, core outcome set protocols, and core outcome sets without reported consensus methods were excluded, as were articles reporting core outcome sets spanning children and adults. Titles and abstracts were first independently reviewed by one author (VH) with a

Confidential: For Review Only

randomly selected 25% double screened by a second author (ER). Full texts of articles meeting the inclusion criteria were then further reviewed, with a randomly selected 50% double screened. Reasons for exclusion were recorded at both stages. Agreement between authors at screening and full text stages was 94% and 82% respectively, and discrepancies were resolved through discussion.

Data extraction and analysis

Study-specific forms were developed, and piloted; and subsequently used by two authors (VH, ER) to independently extract data on: authorship; the core outcome set scope, use, and outcomes listed; and consensus methodology used, including method type, stakeholder numbers, and patient/public involvement. The ICF was used as a framework for coding the outcome data; this provided a common, internationally agreed terminology for naming and describing health outcomes. Outcomes were considered health indicators if they directly related to the child. Outcomes related to care inputs and processes (e.g., resources, length of stay, attendance) were excluded. Outcomes were coded using a published ICF decision tree.[16] For the full mapping, please see the full data set.[17] Article screening was done in EndNote; data extraction in Microsoft Word; and analysis in Microsoft Excel.

RESULTS

We identified 206 records, of which 68 duplicates were removed. A further 86 records were excluded following title and abstract screening, 16 were excluded following full text assessment (Figure 1), and 36 studies were selected for inclusion in full review (Table 2). Selected articles described 36 core outcome sets related to: gastrointestinal conditions (n=9); neurological conditions (n=7); ear, nose, and throat (n=5); orthopaedics (n=3); general paediatrics (n=3); neonatology (n=2); respiratory (n=2); metabolic disease (n=2); and rheumatology, oncology and dentistry specialities (n=1 each) (Table 2). The number of outcomes in a core set ranged from three to 39, with a median of nine.

https://mc.manuscriptcentral.com/bmjpo

Common outcome domains

From the 36 core outcome sets included, 441 outcomes were extracted. Mapping these to the ICF resulted in 22 clusters of outcomes, linked to 25 unique ICF codes (Table 3). Medical diagnoses formed the largest cluster, activity and participation forming the majority of the other larger clusters. Several smaller clusters related to body functions were identified as well as a cluster relating to growth. Personal factors relating to emotional wellbeing were clustered and showed relatively higher commonality. Environmental factors were included in several sets and collectively formed one, large cluster. Table 4 summarises the top 10 clusters by size after medical diagnostic outcomes and shows how many sets each outcome cluster was represented in.

Stakeholder involvement

Table 5 summarises the stakeholder involvement in the three main sections of core outcome set development: (i) generation of an outcome longlist; (ii) consensus process; and (iii) final consensus meeting. The diversity of stakeholders involved varied (Table 5). Five (14%, 5/36) only sought opinion from clinicians/researchers throughout their development, including no parents or children or young people (CYP) at any stage. The remaining thirty-one (86%, 31/36) included parents/caregivers in at least one part of the development, with sixteen including parents/caregivers in both outcome listing and consensus stages. Eleven (31%, 11/36) included CYP in at least one part, with nine (25%, 9/36) including them in both outcome listing and consensus (Table 5). Seven sets related to neonatal or infant populations; three of these were for conditions likely to impact into later childhood (gastroschisis, brachial plexus injury, infants receiving neonatal care) and so could have considered including older CYP. Parent/caregiver and young person stakeholder representation had increased over time (Figure 2).

Attrition rates varied, with a mean of 21% (range 0-63%) for clinicians, 29% (range 0-95%) for studies including parents in more than one round and 19% (range 0-73%) for studies

Confidential: For Review Only

including young people. Seven (19%, 7/36) mitigated concerns around attrition by recruiting new parent or CYP stakeholder groups for subsequent rounds. There was some evidence that patient and public involvement (PPI) impacted attrition rates, with mean stakeholder attrition lower for studies that utilised PPI (Figure 3, Table 5).

Figure 4 shows all 36 core outcome sets, plotted by stakeholder (parents and/or children) involvement in longlisting (x-axis) and the consensus process (y-axis). This figure highlights that the sets with greater stakeholder involvement tended to be those including a smaller number of domains. The bubble sizes represent the number of individual outcome domains included in the final core outcome set. Sets nearest the top right-hand corner are those with higher proportions of involvement at both stages of the process (representing higher proportions of stakeholder involvement). Clustered around zero are those sets with very minimal, or no stakeholder involvement at either stage, and those to the left of the plot represent those that used reviews only for the longlisting stage, or for which data around stakeholder participation was unavailable.

DISCUSSION

We extracted 441 outcome variables from 36 paediatric core outcome sets, and mapped them to 22 outcome clusters of the International Classification of Functioning, Disability and Health (ICF) as well as on environmental factors potentially affecting these outcomes. These 22 areas represent potential child health indicators for measurement in routine and large-scale data. The most common indicator cluster was a child's diagnosis (in 76% of sets). The second most common was pain (in 33% of sets), followed by activity and participation indicators related to self-care, school, personal well-being, community and civic life (all in 31% of sets), communication and social interactions, and mobility (both in 25% of sets). These were followed by body structure and function indicators relating to sleep (22%), mental functions (16%) and growth (in 11% of sets). In addition, three categories of common indicators residing outside the ICF were identified: mortality (in 33% of sets), dental/oral

Confidential: For Review Only

health (in 8%), and quality of life (in 53% of sets). Overall, while there is an in-principle commitment to patient involvement in core outcome set development, the selection and prioritisation of indicators in the included sets were more informed by clinicians and researchers than children, young people or parents who were under-represented.

The present review used a robust search and data extraction strategy, independent double screening and data extraction by two authors, and a published decision tree for the coding of data on the ICF. The search was limited to the COMET initiative registry, which itself is rigerously updated annually, with the update published as a peer-reviewed systematic review. We can therefore be relatively certain that the present study successfully identified articles up to and including the last update of the regustry (December 2019); the inclusion after that relies on ad hoc author registration and so it is possible that later sets were not included. We did not assess the quality of the paediatric core outcome sets as this was not one of the aims on this review.

The outcome clusters identified in the present review align with other, concurrent initiatives. To date, two approaches to selecting indicators and outcomes have dominated: the public health, and the clinical. In the public health approach, coarse indicators (e.g. births, deaths, vaccination rates) are selected and used for national and regional reporting and comparison. One such prominent example is the *State of Child Health* by Royal College of Paediatrics and Child Health (RCPCH).[2] This uses key child health indicators to monitor trends and provide policy recommendation across the United Kingdom. Mortality, weight, oral health and mental health are all included, corresponding to some of the domains in the present review. Another example is the Public Health England (PHE) child health indicators, used to monitor trends and inform policy.[18] A challenge with the current public health indicators and related data are children at highest risk of long-term ill health are not well represented – including children with health conditions or marginalised due to sociodemographic circumstances. They are missed from data collection, or the data does not cover key

Confidential: For Review Only

indicators relevant to them, for example, the PHE data does not include pain or sleep. In contrast, in the clinical approach, highly specialised end points (e.g. particular illness symptoms, treatment side effects) are selected on the basis of their relevance to specific interventions and clinical groups; these indicators and measurement rarely touch upon all children universally. There may be a tacit assumption that missing the highest risk children and young people from the public health data is compensated for by the clinical data. However, this is problematic as it neglects some universally important aspects of child health for the clinical populations, and creates a two-strand system where children are viewed through a binary “typical” versus “clinical” lens which is then translated into segregated policy and decision making. For example, the UK Chief Medical Officers developed separate physical activity guidelines for typically developing and disabled children, in large part based on an argument that there was no compatible data to inform a joint, integrated guideline. Similar concerns apply to other major health areas of obesity, smoking and drinking, and uptake of vaccines.

Arguably, both of the public health and clinical approaches have emerged from measuring what can be measured within the current system and with existing instruments and been heavily influenced by the dominant expert paradigms of public health and clinical medicine. Both approaches can been criticised for overlooking important aspects of health and wellbeing valued by CYP, families, and decision makers – and thus for resulting in data and findings with limited ability to inform decision making. In the present review, we sought to adopt a third perspective to complement these two approaches, namely to identify ways forward for making more visible and measurable some of the important aspects of health that are not yet strongly featured in either of the existing approaches. Our findings here suggest that there indeed are shared, recognisable, universal health indicators that are likely to apply both to public health and clinical enquiries, such as self-care, pain, sleep, and social interactions. These findings converge with, and further add to, the recent report from another

Confidential: For Review Only

international group that, independently and concurrently to us, sought to advance the thinking around child health measurement.[19] That concurrent work retained a healthcare paradigm and focused on existing standardised measurement tools that could be used across diagnostic groups (explicitly excluding health indicators that were deemed not yet measurable through standardised instruments). Similarly to our review, they found universally important health indicators such as survival, growth, pain, school attendance, and social functioning. Collectively, the findings from these two studies provide a strong foundation for the development of a universal, common child health indicator framework that spans traditional discipline and sector boundaries to complement existing core outcome sets for interventions as well as inform routine public health data collection. Developing such approach has the potential to facilitate more integrated, inclusive policy, practice and research across child health by focusing attention to universally important health and wellbeing goals that matter to all children, including children who may be clinically unwell.

To progress a meaningful child health indicator framework will require further, substantial development. Key to this will be the involvement of a wider pool of stakeholders, particularly CYP, in deciding what the key indicators should be and how these are best operationalised for measurement and data collection. The findings from the present review align with those of the most recent COMET annual update[14] that found only 16% of paediatric core sets included direct input from CYP. Whilst this is lower than 31% of sets found in this review, the authors included sets spanning both adult and CYP populations, suggesting that with a broader review strategy CYP inclusion is even poorer. CYP and parents often differ in their priorities to health experts,[4,20,21] and it cannot be assumed that parents hold the same views as children and young people.[22] Therefore, including CYP as well as parents is crucial. There is evidence that CYP both understand and are keen to be involved in the development of health indicator and outcome sets[23] and in neonatal or infant conditions involvement of older children with relevant experiences may be useful. A key lesson from the present review

Confidential: For Review Only

is that those engaging higher numbers of CYP differed from standard delphi methodology, perhaps paying particular attention to adapting the methods. Furthermore, focusing on a manageable number of indicators may facilitate stakeholder involvement.

There are three immediately-actionable recommendations from the present study. First, national data set administrators should consider adding pain and sleep as key health indicators. Second, anyone developing core outcome or indicator sets should ensure they involve children, young people and parents – with adaptations to the methods to make this feasible. Third, clinical evaluators should consider inclusion of key, universally important child health outcomes that may be relevant to their interventions but absent from the current core sets.

FUNDING STATEMENT

Victoria Harbottle, HEE/NIHR ICA Predoctoral Fellow (NIHR300363), and Niina Kolehmainen, HEE/NIHR ICA Senior Clinical Lecturer (NIHR ICA-SCL-2015-01-003) were funded by the NIHR for this research project. The views expressed in this publication are those of the author(s) and not necessarily those of the NIHR, NHS or the UK Department of Health and Social Care.

COMPETING INTERESTS

Victoria Harbottle and Niina Kolehmainen were funded by the NIHR for part of this research project. No further competing interests to declare.

CONTRIBUTORS

VH and NK concieved this project. VH developed the review protocol with input from NK and BA. VH and ER undertook title, abstract and full text screening, and data extraction, and VH and NK undertook outcome coding. All co-authors provided input into the mauscript writing.

Confidential: For Review Only

I, the Submitting Author has the right to grant and does grant on behalf of all authors of the Work (as defined in the below author licence), an exclusive licence and/or a non-exclusive licence for contributions from authors who are: i) UK Crown employees; ii) where BMJ has agreed a CC-BY licence shall apply, and/or iii) in accordance with the terms applicable for US Federal Government officers or employees acting as part of their official duties; on a worldwide, perpetual, irrevocable, royalty-free basis to BMJ Publishing Group Ltd ("BMJ") its licensees and where the relevant Journal is co-owned by BMJ to the co-owners of the Journal, to publish the Work in Archives of Disease in Childhood and any other BMJ products and to exploit all rights, as set out in our licence.

The Submitting Author accepts and understands that any supply made under these terms is made by BMJ to the Submitting Author unless you are acting as an employee on behalf of your employer or a postgraduate student of an affiliated institution which is paying any applicable article publishing charge ("APC") for Open Access articles. Where the Submitting Author wishes to make the Work available on an Open Access basis (and intends to pay the relevant APC), the terms of reuse of such Open Access shall be governed by a Creative Commons licence – details of these licences and which Creative Commons licence will apply to this Work are set out in our licence referred to above.

Confidential: For Review Only

REFERENCES

1. Jacob CM, Baird J, Barker M, et al. The importance of a life course approach to health: chronic disease risk from preconception through adolescence and adulthood. *Geneva: WHO* 2017
2. Royal College of Paediatrics and Child Health. State of Child Health 2020 [Available from: https://stateofchildhealth.rcpch.ac.uk/.
3. United Nations. The Global Strategy for Women's, Children's and Adolescents' Health (2016-2030). 2015
4. Morris C, Janssens A, Shilling V, et al. Meaningful health outcomes for paediatric neurodisability: Stakeholder prioritisation and appropriateness of patient reported outcome measures. *Health Qual Life Outcomes* 2015;13:87. doi: 10.1186/s12955-015-0284-7 [published Online First: 2015/06/26]
5. Meregaglia M, Ciani O, Banks H, et al. A scoping review of core outcome sets and their 'mapping' onto real-world data using prostate cancer as a case study. *BMC Med Res Methodol* 2020;20(1):41. doi: 10.1186/s12874-020-00928-w [published Online First: 2020/02/28]
6. Kirkham JJ, Davis K, Altman DG, et al. Core Outcome Set-STAndards for Development: The COS-STAD recommendations. *PLoS Med* 2017;14(11):e1002447. doi: 10.1371/journal.pmed.1002447 [published Online First: 2017/11/18]
7. Arksey H, O'Malley L. Scoping studies: towards a methodological framework. *International journal of social research methodology* 2005;8(1):19-32.
8. Peters MDJ, Marnie C, Tricco AC, et al. Updated methodological guidance for the conduct of scoping reviews. *JBI Evidence Synthesis* 2020;18(10) doi: http://dx.doi.org/10.11124/JBIES-20-00167
9. Tricco AC, Lillie E, Zarin W, et al. PRISMA Extension for Scoping Reviews (PRISMA-ScR): Checklist and Explanation. *Ann Intern Med* 2018;169(7):467-73. doi: 10.7326/M18-0850 [published Online First: 2018/09/05]
10. Chien PF, Khan KS, Siassakos D. Registration of systematic reviews: PROSPERO. *BJOG: An International Journal of Obstetrics & Gynaecology* 2012;119(8):903-05. doi: https://doi.org/10.1111/j.1471-0528.2011.03242.x
11. World Health O. International classification of functioning, disability and health : ICF. Geneva: World Health Organization, 2001.
12. The COMET Initiative. [Available from: https://www.comet-initiative.org/ accessed January 2020.
13. Gargon E, Gorst SL, Williamson PR. Choosing important health outcomes for comparative effectiveness research: 5th annual update to a systematic review of core outcome sets for research. *PLoS One* 2019;14(12):e0225980. doi: 10.1371/journal.pone.0225980 [published Online First: 2019/12/13]
14. Gargon E, Gorst SL, Matvienko-Sikar K, et al. Choosing important health outcomes for comparative effectiveness research: 6th annual update to a systematic review of core outcome sets for research. *PLOS ONE* 2021;16(1):e0244878. doi: 10.1371/journal.pone.0244878
15. Sinha I, Jones L, Smyth RL, et al. A Systematic Review of Studies That Aim to Determine Which Outcomes to Measure in Clinical Trials in Children. *PLoS Medicine* 2008;5(4):e96. doi: 10.1371/journal.pmed.0050096
16. Cieza A, Fayed N, Bickenbach J, et al. Refinements of the ICF Linking Rules to strengthen their potential for establishing comparability of health information. *Disability and Rehabilitation* 2019;41(5):574-83. doi: 10.3109/09638288.2016.1145258
17. Harbottle V, Kolehmainen N. Paediatric Core Outcome Sets Review Coding. doi: 10.25405/data.ncl.19589644 Online data set, Last Accessed April 2022.
18. Public Health England. Child Health Profiles 2021 [Available from: https://fingertips.phe.org.uk/profile/child-health-profiles accessed January 2021.

Confidential: For Review Only

19. Algurén B, Ramirez JP, Salt M, et al. Development of an international standard set of patient-centred outcome measures for overall paediatric health: a consensus process. *Archives of Disease in Childhood* 2021;106(9):868-76. doi: 10.1136/archdischild-2020-320345
20. Singendonk MMJ, Rexwinkel R, Steutel NF, et al. Development of a Core Outcome Set for Infant Gastroesophageal Reflux Disease. *Journal of Pediatric Gastroenterology and Nutrition* 2019;68(5):655-61. doi: 10.1097/mpg.0000000000002245
21. Sinha IP, Gallagher R, Williamson PR, et al. Development of a core outcome set for clinical trials in childhood asthma: a survey of clinicians, parents, and young people. *Trials* 2012;13(1):103. doi: 10.1186/1745-6215-13-103
22. Zeevenhooven J, Rexwinkel R, Van Berge Henegouwen VWA, et al. A Core Outcome Set for Clinical Trials in Pediatric Functional Abdominal Pain Disorders. *The Journal of Pediatrics* 2020;221:115-22.e5. doi: 10.1016/j.jpeds.2020.02.032
23. Sherratt FC, Bagley H, Stones SR, et al. Ensuring young voices are heard in core outcome set development: international workshops with 70 children and young people. *Res Involv Engagem* 2020;6:19. doi: 10.1186/s40900-020-00202-9 [published Online First: 2020/05/12]
24. Allin BSR, Hall NJ, Ross AR, et al. Development of a gastroschisis core outcome set. *Arch Dis Child Fetal Neonatal Ed* 2019;104(1):F76-F82. doi: 10.1136/archdischild-2017-314560 [published Online First: 2018/03/16]
25. Allori AC, Kelley T, Meara JG, et al. A Standard Set of Outcome Measures for the Comprehensive Appraisal of Cleft Care. *The Cleft Palate-Craniofacial Journal* 2017;54(5):540-54. doi: 10.1597/15-292
26. Balakrishnan K, Bauman N, Chun RH, et al. Standardized Outcome and Reporting Measures in Pediatric Head and Neck Lymphatic Malformations. *Otolaryngology–Head and Neck Surgery* 2015;152(5):948-53. doi: 10.1177/0194599815577602
27. Balakrishnan K, Sidell DR, Bauman NM, et al. Outcome measures for pediatric laryngotracheal reconstruction: International consensus statement. *The Laryngoscope* 2019;129(1):244-55. doi: 10.1002/lary.27445
28. Bösch F, Zeltner NA, Baumgartner MR, et al. Key patient-reported outcomes in children and adolescents with intoxication-type inborn errors of metabolism: an international Delphi-based consensus. *Orphanet Journal of Rare Diseases* 2022;17(1) doi: 10.1186/s13023-022-02183-2
29. Butler DP, De la Torre A, Borschel GH, et al. An International Collaborative Standardizing Patient-Centered Outcome Measures in Pediatric Facial Palsy. *JAMA Facial Plast Surg* 2019;21(5):351-58. doi: 10.1001/jamafacial.2019.0224
30. Crudgington H, Rogers M, Bray L, et al. Core Health Outcomes in Childhood Epilepsy (CHOICE): Development of a core outcome set using systematic review methods and a Delphi survey consensus. *Epilepsia* 2019;60(5):857-71. doi: 10.1111/epi.14735
31. Damhuis SE, Bloomfield FH, Khalil A, et al. A Core Outcome Set and minimum reporting set for intervention studies in growth restriction in the NEwbOrN: the COSNEON study. *Pediatric Research* 2020 doi: 10.1038/s41390-020-01119-5
32. de Graaf H, Sukhtankar P, Arch B, et al. Duration of intravenous antibiotic therapy for children with acute osteomyelitis or septic arthritis: a feasibility study. *Health Technol Assess* 2017;21(48):1-164. doi: 10.3310/hta21480 [published Online First: 2017/09/02]
33. Gilchrist FJ, Ali I, Brodlie M, et al. Developing a core outcome set for children with protracted bacterial bronchitis. *ERJ Open Research* 2020;6(1):00344-2019. doi: 10.1183/23120541.00344-2019
34. Haeusler GM, Phillips RS, Lehrnbecher T, et al. Core outcomes and definitions for pediatric fever and neutropenia research: A consensus statement from an international panel. *Pediatric Blood & Cancer* 2015;62(3):483-89. doi: 10.1002/pbc.25335

Confidential: For Review Only

35. Harman NL, Bruce IA, Kirkham JJ, et al. The Importance of Integration of Stakeholder Views in Core Outcome Set Development: Otitis Media with Effusion in Children with Cleft Palate. *PLOS ONE* 2015;10(6):e0129514. doi: 10.1371/journal.pone.0129514
36. Heiligenhaus A, Foeldvari I, Edelsten C, et al. Proposed outcome measures for prospective clinical trials in juvenile idiopathic arthritis–associated uveitis: A consensus effort from the multinational interdisciplinary working group for uveitis in childhood. *Arthritis Care & Research* 2012;64(9):1365-72. doi: 10.1002/acr.21674
37. Joachim KC, Farid-Kapadia M, Butcher NJ, et al. Core outcome set for children with neurological impairment and tube feeding. *Developmental Medicine & Child Neurology* 2020;62(2):201-06. doi: 10.1111/dmcn.14326
38. Karas J, Ashkenazi S, Guarino A, et al. A core outcome set for clinical trials in acute diarrhoea. *Archives of Disease in Childhood* 2015;100(4):359-63. doi: 10.1136/archdischild-2014-307403
39. Kuizenga-Wessel S, Steutel NF, Benninga MA, et al. Development of a core outcome set for clinical trials in childhood constipation: a study using a Delphi technique. *BMJ Paediatr Open* 2017;1(1):e000017. doi: 10.1136/bmjpo-2017-000017 [published Online First: 2018/04/11]
40. Leo DG, Jones H, Murphy R, et al. The outcomes of Perthes' disease. *The Bone & Joint Journal* 2020;102-B(5):611-17. doi: 10.1302/0301-620X.102B5.BJJ-2020-0072
41. Liu PZ, Ismail-Koch H, Stephenson K, et al. A core outcome set for research on the management of otitis media with effusion in otherwise-healthy children. *International Journal of Pediatric Otorhinolaryngology* 2020;134:110029. doi: 10.1016/j.ijporl.2020.110029
42. Marson BA, Manning JC, James M, et al. Development of the CORE-Kids core set of outcome domains for studies of childhood limb fractures. *Bone Joint J* 2021;103-b(12):1821-30. doi: 10.1302/0301-620x.103b.Bjj-2020-2321.R2 [published Online First: 2021/08/21]
43. Matvienko-Sikar K, Griffin C, Kelly C, et al. A core outcome set for trials of infant-feeding interventions to prevent childhood obesity. *International Journal of Obesity* 2020 doi: 10.1038/s41366-020-0538-2
44. Murugupillai R, Ranganathan SS, Wanigasinghe J, et al. Development of outcome criteria to measure effectiveness of antiepileptic therapy in children. *Epilepsy & Behavior* 2018;80:56-60. doi: 10.1016/j.yebeh.2017.12.011
45. Nabbout R, Auvin S, Chiron C, et al. Development and content validation of a preliminary core set of patient- and caregiver-relevant outcomes for inclusion in a potential composite endpoint for Dravet Syndrome. *Epilepsy Behav* 2018;78:232-42. doi: 10.1016/j.yebeh.2017.08.029 [published Online First: 2017/11/08]
46. Palermo TM, Walco GA, Paladhi UR, et al. Core outcome set for pediatric chronic pain clinical trials: results from a Delphi poll and consensus meeting. *Pain* 2021;162(10):2539-47. doi: 10.1097/j.pain.0000000000002241
47. Pease A, Goodenough T, Borwick C, et al. Development of a core outcome set for evaluative research into paediatric cerebral visual impairment (CVI), in the UK and Eire. *BMJ Open* 2021;11(9):e051014. doi: 10.1136/bmjopen-2021-051014
48. Pondaag W, Malessy MJA. Outcome assessment for Brachial Plexus birth injury. Results from the iPluto world-wide consensus survey. *Journal of Orthopaedic Research* 2018;36(9):2533-41. doi: 10.1002/jor.23901
49. Pugliese M, Tingley K, Chow A, et al. Core Outcome Sets for Medium-Chain Acyl-CoA Dehydrogenase Deficiency and Phenylketonuria. *Pediatrics* 2021;148(2) doi: 10.1542/peds.2020-037747 [published Online First: 2021/07/17]
50. Rexwinkel R, Van Berge Henegouwen VWA, Singendonk MMJ, et al. Development of a Core Outcome Set for Children Aged 1-18 Years with Gastroesophageal Reflux Disease. *The Journal of Pediatrics* 2022 doi: 10.1016/j.jpeds.2022.01.043
51. Smaïl-Faugeron V, Fron Chabouis H, Durieux P, et al. Development of a Core Set of Outcomes for Randomized Controlled Trials with Multiple Outcomes – Example of Pulp Treatments of

Confidential: For Review Only

Primary Teeth for Extensive Decay in Children. *PLoS ONE* 2013;8(1):e51908. doi: 10.1371/journal.pone.0051908
52. Steutel NF, Benninga MA, Langendam MW, et al. Developing a core outcome set for infant colic for primary, secondary and tertiary care settings: a prospective study. *BMJ Open* 2017;7(5):e015418. doi: 10.1136/bmjopen-2016-015418
53. Topjian Alexis A, Scholefield Barnaby R, Pinto Neethi P, et al. P-COSCA (Pediatric Core Outcome Set for Cardiac Arrest) in Children: An Advisory Statement From the International Liaison Committee on Resuscitation. *Circulation* 2020;0(0):CIR.0000000000000911. doi: 10.1161/CIR.0000000000000911
54. Webbe JWH, Duffy JMN, Afonso E, et al. Core outcomes in neonatology: development of a core outcome set for neonatal research. *Archives of Disease in Childhood - Fetal and Neonatal Edition* 2020;105(4):425. doi: 10.1136/archdischild-2019-317501

Confidential: For Review Only

TABLES AND FIGURES

Table 1: COMET Database Search Criteria

Health Area-disease category<br>Child Health<br>AND<br>Publication year<br>2008 onwards<br>AND<br>Published/unpublished<br>Published
OR
Minimum age<br>0<br>AND<br>Maximum age<br>18<br>AND<br>Publication year<br>2008 onwards<br>AND<br>Published/unpublished<br>Published

Table 2: Overview of included paediatric core outcome sets

Author/ Year	Scope of set			Size of Set
	Age range	Disease group	Use	
Alin et al, 2019 [24]	Birth upwards	Gastroschisis	Research	8
Allori et al, 2017 [25]	'Child'	Cleft lip/palate	Research + clinical (benchmarking)	22
Balakrishnan et al, 2015 [26]	'Paediatric'	Head and neck lymphatic malformations	Research	31
Balakrishnan et al, 2019 [27]	'Paediatric'	Laryngotracheal reconstruction	Research and clinical	8
Bösch et al, 2022 [28]	'Paediatric'	Intoxication-type inborn errors of metabolism	Research	17
Butler et al, 2019 [29]	0-18 yrs	Facial palsy	Quality of care	20
Crudgington et al, 2019 [30]	5-16 yrs	Rolandic epilepsy	Research but mentions clinical use (audit, SE)	39
Damhuis et al, 2020 [31]	Birth	Growth restriction in newborns	Research	19
De Graaf et al, 2017 [32]	'Children'	Acute osteomyelitis and septic arthritis	Research-specific RCT being planned	11

Confidential: For Review Only

Gilchrist et al, 2020 [33]	'Children'	Protracted bacterial bronchitis	Research	6
Haeusler et al, 2015 [34]	'Children'	Fever and neutropenia	Research	10
Harman et al, 2015 [35]	<18 yrs	Otitis media with effusion (in cleft palate)	Research	9
Heiligenhaus et al, 2012 [36]	Implied <16 yrs	JIA-associated uveitis	Research	7
Joachim et al, 2020 [37]	'Children'	Neurological impairment and enteral tube feeding	Research	12
Karas et al, 2015 [38]	'Paediatric'	Acute diarrhoea	Research	5
Kuizenga-Wessel et al, 2017 [39]	0-18 yrs	Functional constipation	Research	8
Leo et al, 2020 [40]	'Children'	Perthes disease	Research and clinical practice	14
Liu et al, 2020 [41]	<12 yrs	Otitis media with effusion (in otherwise healthy children)	Research	9
Marson et al, 2021 [42]	5-16 yrs	Limb fractures	Research	8 + 1 additional for upper or lower limb
Matvienko-Sikar et al, 2020 [43]	<1 yr	Infant feeding for the prevention of childhood obesity	Research	25
Murugupillai et al, 2018 [44]	Two sets: Pre-school <6 yrs School-age 6-18 yrs	Effectiveness of anti-epilepsy therapy in children	Research	8
Morris et al, 2015 [4]	'Children'	Neurodisability	Informing development of NHS Outcomes Framework	12
Nabbout at el, 2018 [45]	2-18 yrs	Dravet Syndrome	Research	5
Palermoa et al, 2021 [46]	'Paediatric'	Chronic pain	Research	4 mandatory, 3 optional
Pease et al, 2021 [47]	0-18 yrs	Paediatric cerebral visual impairment	Research	27
Pondaag and Malessy, 2018 [48]	Not stated	Brachial plexus birth injury	Research and clinical-universal dataset	3
Pugliese et al, 2021 [49]	<12 yrs	Medium-chain acyl-coenzyme A dehydrogenase deficiency (MCAD) and	Research	MCAD- 8 PKU- 9

Confidential: For Review Only

		phenylketonuria (PKU)		
Rexwinkle et al, 2022 [50]	1-18 yrs	Gastroesophageal refulx	Research and clinical guidelines	9
Sherratt et al, 2020	<18 yrs	Uncomplicated appendicitis	Research	14
Singendonk et al, 2019 [20]	'Infants'	Infant gastroesophageal reflux disease	Research	9
Sinha et al, 2012 [21]	<5 yrs and 5-18 considered separately	Chronic childhood asthma	Research, pilot	4
Smail-faugeron et al, 2013 [51]	Children with primary teeth	Pulp treatment for primary teeth	Research	5
Steutel et al, 2017 [52]	'Infants'	Infant colic	Research	6
Topjian Alexis et al, 2020 [53]	'Children'	Cardiac arrest	Research	5
Webbe et al, 2020 [54]	Neonates	Neonatology	Research	12
Zeevenhooven et al, 2020 [22]	'Paediatric'	Functional abdominal pain disorders	Research	8

Confidential: For Review Only

Table 3: Overview of coded and grouped outcomes extracted from paediatric core outcome sets.

Cluster (n total)	ICF code	n	Examples
Communication (n=18)	Voice and speech functions (b3)	7	Articulation, overall speech, speech ability
	Communication (d3)	11	Receptive language skills, listening skills, receptive communication
Self-care (n= 18)	Eating (d550) Drinking (incl. breast feeding) (d560)	9	Breast feeding, child self/assisted feeding, feeding difficulties
	Self-care (d5)	9	Activities of daily living, toileting, safety
Mobility, movement, and related structures (n=19)	Mobility (d4)	10	Gross motor, fine motor, motor impairment, mobility
	Mobility of joint functions (b710)	4	Hip mobility, passive range of movement, active range of movement
	Structures relating to movement (s7)	5	Limb deformity, femoral head shape, arthritic changes to hip
School and learning (n= 19)	School education (d820)	11	School attendance, engagement in school life,
	Learning and applying knowledge (d1)	8	Literacy, academic attainment, school performance
Social (n= 18)	Interpersonal interactions and relationships (d7)	11	Friendships, relationship with parents/siblings, psychosocial development
	Managing own behaviour (d520)	7	Behavioural concerns, behaviour
Community, play and civic life (n= 14)	Community, social and civic life (d9)	14	Sport participation, time away from activities, social activities, play
Growth (n= 12)	Growth maintenance functions (b560)	6	Head circumference, length, adequate growth
	Weight maintenance functions(b530)	6	Weight, weight gain over time, body composition
Pain (n= 17)	Pain (b280-b289)	17	Pain, abdominal pain,
Mental functions	General mental functions (b1)	5	General cognitive ability, cognitive impairment, psychosocial development

Confidential: For Review Only

(n=11)	Specific mental functions (b140-199)	6	Memory, executive function
Sleep (n= 11)	Sleep (b134)	11	Duration of sleep, awakenings from sleep, sleep quality
Function of senses (n= 11)	Hearing functions (b230)	4	Hearing, hearing impairment,
	Seeing functions (b210)	7	Visual acuity, visual performance
Digestive system functions (n= 8)	Digestive functions (b515)	4	Bowel obstruction, vomiting
	Defecation functions (b525)	4	Defecation pattern, stool consistency, defecation frequency
Respiratory functions (n= 4)	Respiration functions (b440)	3	Breathing difficulties, airway compromise, airway obstruction
	Additional respiratory functions (b450)	1	Cough
Skin (n= 2)	Structure of areas of skin (s810)	2	Skin involvement, skin necrosis
Environmental factors (n=33)	e	33	Family life, mother's knowledge of how to offer food, family stress, family quality of life
Medical diagnosis (n=84)	N/A	84	Sepsis, liver disease, infection, seizure
Quality of life (n= 19)	N/A	19	
Death (n= 13)	N/A	13	Death, mortality, all cause mortality, infection related mortality
Ear, nose, throat and dental (n= 12)	N/A	12	Dental health, oral health, occlusion, facial profile, smile, pathologic root resorption
Personal factors relating to emotional wellbeing (n= 17)	N/A	17	Feelings, self-esteem, feelings about having epilepsy, fear of seizures, emotional wellbeing
Other personal factors (n= 10)	N/A	10	Concealment of condition, psychosocial development, attitude towards disease

Confidential: For Review Only

Table 4: Top 10 outcome clusters by size (excluding medical diagnostic outcomes)

Cluster	Number of outcomes mapped to cluster (n/441)	Number of sets represented in (n/n, %)
Environmental	33	10/36, 28%
School and learning	19	11/38, 31%
Mobility, movement, and related structures	19	9/36, 25%
Quality of life	19	19/36, 53%
Communication	18	9/36, 25%
Self-care	18	11/36, 31%
Social	18	7/36, 19%
Pain	17	12/36, 33%
Personal factors relating to emotional wellbeing	17	11/36, 31%
Community, play and civic life	14	11/36, 31%

Confidential: For Review Only

Table 5: Summary of stakeholder group involvement in paediatric core outcome set development

**Author/year**	**Outcome Listing**	**Stakeholders included (numbers)**				**Consensus method**	**Stakeholders included (numbers participating across all rounds)**				**Final Consensus Meeting**		**Steering Group Explicitly Mentioned**		
		**Clinicians**	**Parents**	**Young People**	**Other**		**Clinicians**	**Parents**	**Young People**	**Other**	**Y/N**	**P/CYP Included?**	**Y/N**	**P/CYP Included?**	**Other Groups**
Alin et al, 2019	Systematic review	-	-	-	-	Delphi	49	22	0	0	▯	Parents only	▯	N	
Allori et al, 2017	Systematic review, qualitative (clinicians only) - interviews, details not published	-	-	-	-	Modified delphi	28	2	1	0	X	-	▯	▯	X
Balakrishnan et al, 2015	Survey	9	0	0	0	Modified delphi	9	0	0	0	X	-	X	-	X
Balakrishnan et al, 2019	Survey	33	0	0	0	Delphi	33	0	0	0	X	-	X	-	X
Bösch et al, 2022	Systematic review, qualitative focus groups	0	26	19	0	Survey (2 rounds)	30	24	9	0	▯	▯	X	-	Survey piloted with stakeholder s including CYP
Butler et al, 2019	Review, communicati	18	3	0	0	Modified delphi	18	3	0	0	X	-	▯	X	Patient advisory

Confidential: For Review Only

	on with patient advisory groups														groups consulted-adults with paediatric-onset palsy
Crudgington et al, 2019	Systematic review	-	-	-	-	Delphi	61	16	3		□	□	X	X	Consulted young person's advisory group
Damhuis et al, 2020	Systematic review	-	-	-	-	Delphi	33	14	0	0	□	Parents only	□	□	X
De Graaf et al, 2017	Systematic review	-	-	-	-	Delphi	97	0	0	0	□	Parents + young person represen tative	X	-	X
Gilchrist et al, 2020	Systematic review, qualitative interviews with parets, clinician survey	20	16	0	0	Delphi	51	0	0	0	□	Parents only	X	-	X
Haeusler et al, 2015	Review of guidelines and consensus statements	-	-	-	-	Delphi	33	3	0	0	X	-	□	X	X
Harman et al, 2015	Systematic review, qualitative interviews	-	43	22	-	Modified delphi	75	35	8	8	□	X	□	□	Study advisory group-invited 1 x CYP but withdrew

Confidential: For Review Only

															Sought advice from diagnosis specific YPAG
Heiligenhaus et al, 2012	Systematic review	-	-	-	-	Delphi, nominal group	16	0	0	0	□	X	X	-	X
Joachim et al, 2020	Systematic review	-	-	-	-	Delphi	15	3	0	0	□	Invited, unable to attend, further remote validatio n with parents	□	X	X
Karas et al, 2015	Systematic review, survey	66	31	0	4	Delphi	64	32	0	0	X	-	□	X	X
Kuizenga-Wessel et al, 2017	Survey	10 9	16 5	50		Modified delphi	50	80	50	0	□	X	X	X	X
Leo et al, 2020	Systematic review, qualitative interviews,	0	18	12	0	Delphi	36	46	18	0	□	Parents only	X	-	X
Lui et al, 2020	Systematic review, qualitative focus groups, examination of tools used in trials currently	0	3	0	0	Survey	81	53	0	0	X	-	X	-	X

Confidential: For Review Only

Marson et al, 2021	Systematic review, qualitative interviews	0	20	20	0	Delphi	111	19	0	15	□	□	X	-	Patient, parent and public advisory group consulted
Matvienko-Sikar et al, 2020	Systematic review and stakeholder meeting	12	0	0	0	Delphi	75	2	0	2	□	Parents only	X	-	X
Morris et al, 2015	Systematic review, qualitative focus groups and interviews, delphi-clinicians	191	53	54	0	Nominal group	7	5	3	0	X	-	□	□	Pilot to refine young person participation methods
Murugupillai et al, 2018	Survey	32	50	15	0	Delphi	29	42	0	0	X	-	X	-	X
Nabbout at el, 2018	Qualitative semi structured interviews informed by systematic review	4	7	0	0	Modified delphi	7	0	0	0	X	-	X	-	X
Palermoa et al, 2021	Survey	52	90	93	0	Modified delphi	44	85	86	0	□	X	□	X	Patient advisory group consulted
Pease et al, 2021	Review, qualitative interviews	0	18	6	0	Delphi	52	28	0	0	□	Parents only	X	-	2 x patient involvement meetings with

Confidential: For Review Only

															families, parent and CYP involvement in study design
Pondaag and Malessy, 2018	Systematic review then Survey - binary yes/no for inclusion	69	0	0	0	Survey	59	0	0	0	*X*	-	X	-	X
Pugliese et al, 2021	Systematic review	-	-	-	-	Delphi	16	37	0	0	□	Parents only	X	-	2 patient partner investigators with lived experience, led family advisory forum
Rexwinkle et al, 2022	Systematic review, health professionals survey and parent/CYP questionnaire	118	146	69	0	Delphi	80	130	77	0	□	X	X	-	X
Sherratt et al, 2020	Systematic review + qualitative interviews (parents and CYP) (interviews embedded in unpublished					Delphi	110	32	3	0	□	□	X	-	Parents and young persons study advisory group

Confidential: For Review Only

	study so no no. available)														
Singendonk et al, 2019	Survey	125	139	0	0	Delphi	89	127	0	0	□	X	X	-	X
Sinha et al, 2012	Survey	46	28	11	0	Delphi - clinicians Survey- parents and CYP	43	50	0	0	X	-	X	-	Pilot phase tested study materials with parents and young people
Smail-faugeron et al, 2013	Systematic review + stakeholder meeting	6	0	0	0	Delphi	52	0	0	0	X	-	X	-	X
Steutel et al, 2017	Systematic review, Survey	133	55	0	0	Delphi	54	43	0	0	□	X	□	X	X
Topjian Alexis et al, 2020	Used adult set, + suggestions from steering committee	18	0	0	0	Delphi	68	6	0	0	X	-	□	X	X
Webbe et al, 2020*	Systematic review x 2 (trials and qual)	-	-	-	-	Delphi	130	53	0	0	□	□	□	□	X
Zeevenhooven et al, 2020	Systematic review + Survey	152	103	50	0	Delphi	104	102	53	0	□	X	X	X	X

Confidential: For Review Only

Table 6: Attrition of different stakeholder groups throughout consensus rounds

Author/year	No. of rounds	Attrition first to last rounds (%)			Notes
		Clinicians	Parents	Young people	
Alin et al, 2019	3	18	48	-	Attempt to recruit young people, unsuccessful
Allori et al, 2017	7	0	0	0	
Balakrishnan et al, 2015	3	33	-	-	
Balakrishnan et al, 2019	2	0	-	-	
Bösch et al, 2022	2	17	11	0	
Butler et al, 2019	7 x 2 round delphi	Attrition rates not recorded			
Crudgington et al, 2019	2	20	30	0	
Damhuis et al, 2020	3	3	26	-	
De Graaf et al, 2017	2	33	-	-	
Gilchrist et al, 2020	2	22	-	-	
Haeusler et al, 2015	4	15	25	-	
Harman et al, 2015	3	30	-	-	Parents and young people completed 1 round survey only
Heiligenhaus et al, 2012	2 (1 x delphi, 1 x nominal group	12	-	-	
Joachim et al, 2020	1	-	-	-	
Karas et al, 2015	2	9	-	-	Different group of parents invited to round 2
Kuizenga-Wessel et al, 2017	2	-	-	-	Different stakeholder groups of invited to round 2
Leo et al, 2020	2	22	49	33	
Lui et al, 2020	1	-	-	-	
Marson et al, 2021	3	31	27	-	
Matvienko-Sikar et al, 2020	3	63	95	0	
Morris et al, 2015	1	-	-	-	1 round nominal group
Murugupillai et al, 2018	2	9	16	-	Young people completed 1 round survey only
Nabbout at el, 2018	2	13	-	-	

Confidential: For Review Only

Palermoa et al, 2021	2	15	6	8	
Pease et al, 2021	2	35	30	-	
Pondaag and Malessy, 2018	3	14	-	-	
Pugliese et al, 2021	3	48	38	-	
Rexwinkle et al	1	-	-	-	
Sherratt et al, 2020	3	30	44	73	
Singendonk et al, 2019	2	-	-	-	Further clinician recruitment for round 2 Different group of parents invited to round 2
Sinha et al, 2012	2	6	-	-	Different group of parents invited to round 2, no young people invited to round 2
Smaïl-Faugeron et al, 2013	3	16	-	-	
Steutel et al, 2017	2	59	-	-	Different group of parents invited to round 2
Topjian Alexis et al, 2020	2	18	0	-	
Webbe et al, 2020	3	13	52	-	Parents and young people combined in one group
Zeevenhooven et al, 2020	2	32	-	-	Different group of parents and young people invited to round 2

Confidential: For Review Only

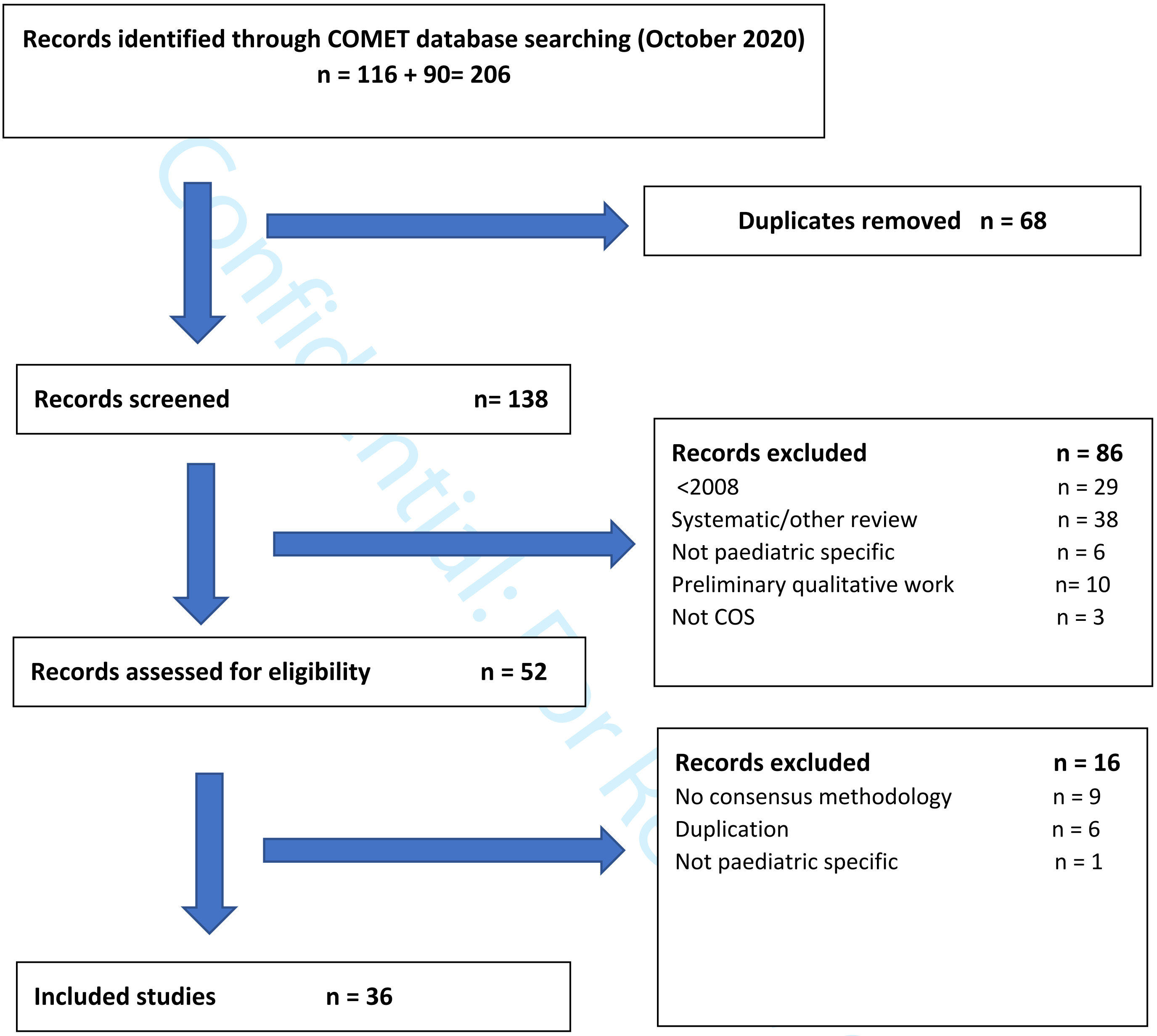

Figure 1: Summary of screening strategy

Confidential: For Review Only

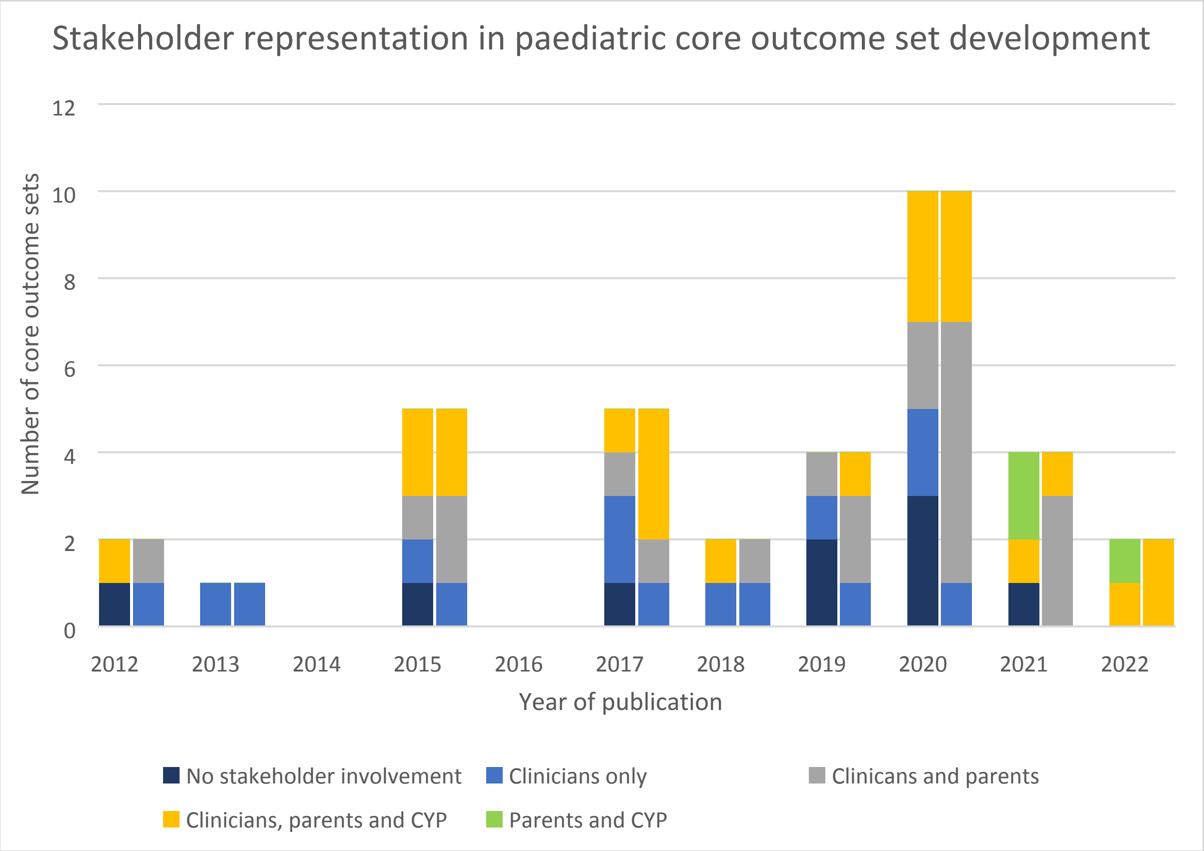

Figure 2: Stakeholder groups involved in (i) outcome long listing and (ii) consensus process by year

Left hand bar represents outcome long listing, right hand bar represents consensus process

For Review Only

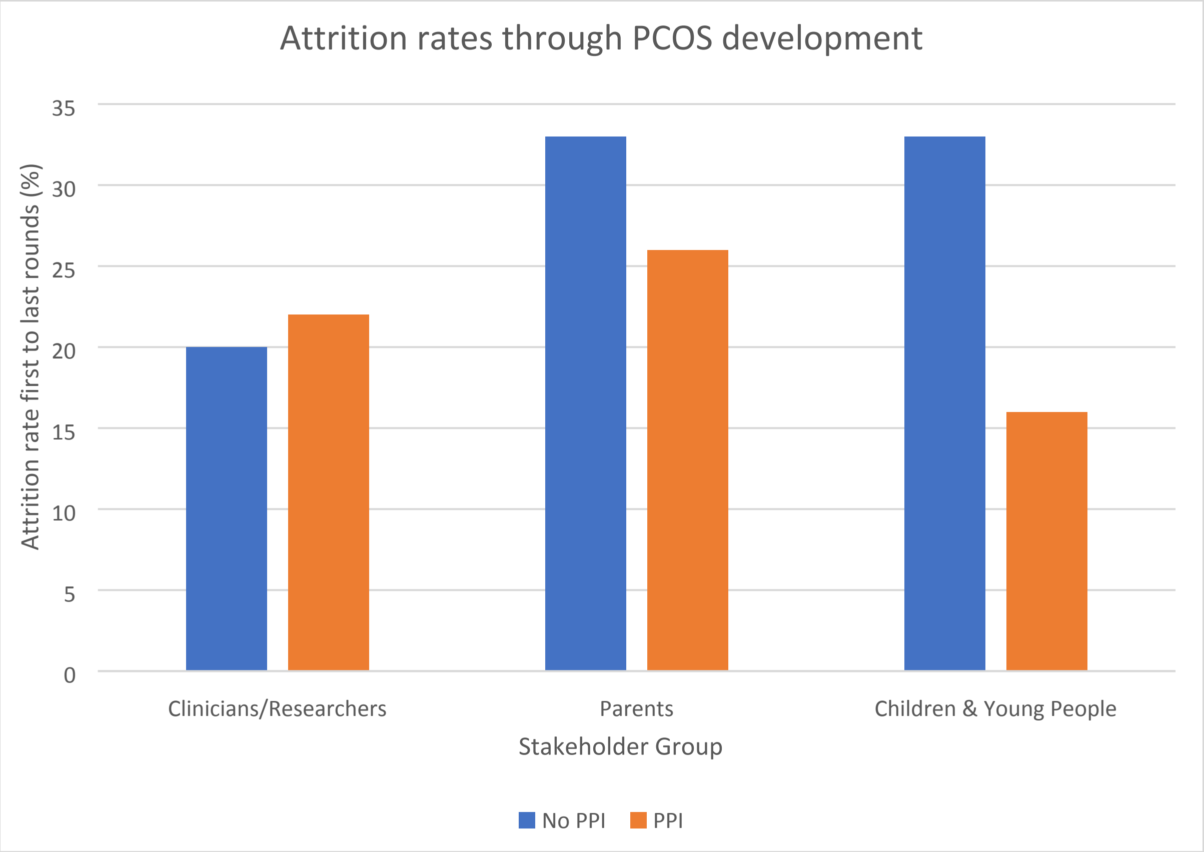

Figure 3: Mean attrition throughout paediatric core outcome set development by stakeholder group, comparing those with PPI involvement to those without.

For Review Only

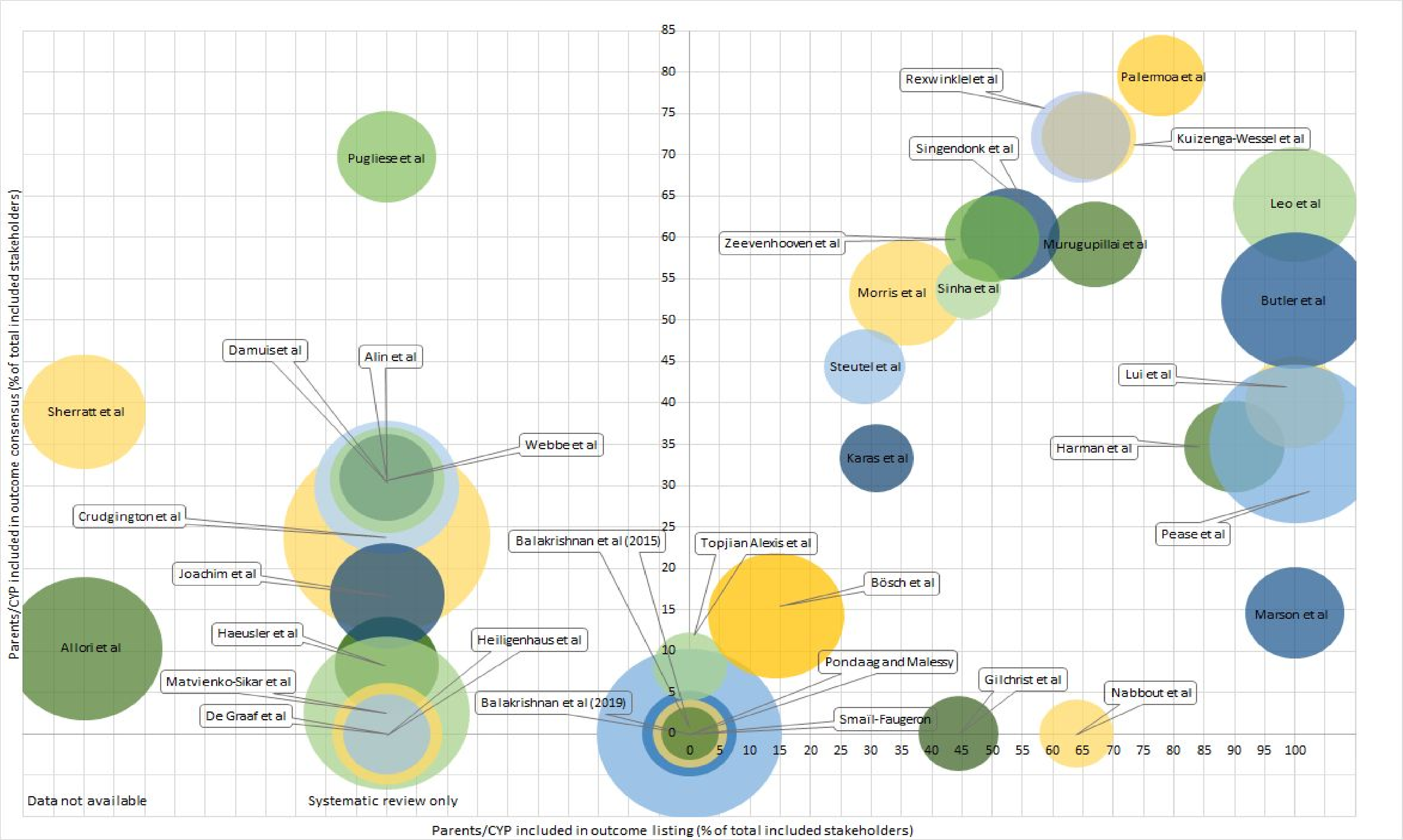

Figure 4: Stakeholder involvement in outcome long listing and outcome consensus for all paediatric core outcome sets. Bubble size represents final paediatric core outcome set size

BMJ Paediatrics Open

Identifying common health indicators from paediatric core outcome sets: a systematic review with narrative synthesis using the WHO International Classification of Functioning, Health and Disability

Journal:	*BMJ Paediatrics Open*
Manuscript ID	bmjpo-2022-001537.R1
Article Type:	Review
Date Submitted by the Author:	08-Jul-2022
Complete List of Authors:	Harbottle, Victoria; Great North Children's Hospital, Rehabilitation Department; Newcastle University, Population Health Sciences Institute<br>Arnott, Bronia; Newcastle University, Population Health Sciences Institute<br>Gale, Chris; Imperial College London, Academic Neonatal Medicine<br>Rowen, Elizabeth; Great North Children's Hospital, Rehabilitation Department<br>Kolehmainen, Niina; Newcastle University, Population Health Sciences Institute; Newcastle Upon Tyne Hospitals NHS Foundation Trust,
Keywords:	Data Collection, Adolescent Health, Epidemiology

SCHOLARONE™
Manuscripts

I, the Submitting Author has the right to grant and does grant on behalf of all authors of the Work (as defined in the below author licence), an exclusive licence and/or a non-exclusive licence for contributions from authors who are: i) UK Crown employees; ii) where BMJ has agreed a CC-BY licence shall apply, and/or iii) in accordance with the terms applicable for US Federal Government officers or employees acting as part of their official duties; on a worldwide, perpetual, irrevocable, royalty-free basis to BMJ Publishing Group Ltd ("BMJ") its licensees and where the relevant Journal is co-owned by BMJ to the co-owners of the Journal, to publish the Work in this journal and any other BMJ products and to exploit all rights, as set out in our licence.

The Submitting Author accepts and understands that any supply made under these terms is made by BMJ to the Submitting Author unless you are acting as an employee on behalf of your employer or a postgraduate student of an affiliated institution which is paying any applicable article publishing charge ("APC") for Open Access articles. Where the Submitting Author wishes to make the Work available on an Open Access basis (and intends to pay the relevant APC), the terms of reuse of such Open Access shall be governed by a Creative Commons licence – details of these licences and which Creative Commons licence will apply to this Work are set out in our licence referred to above.

Other than as permitted in any relevant BMJ Author's Self Archiving Policies, I confirm this Work has not been accepted for publication elsewhere, is not being considered for publication elsewhere and does not duplicate material already published. I confirm all authors consent to publication of this Work and authorise the granting of this licence.

Confidential: For Review Only

Identifying common health indicators from paediatric core outcome sets: a systematic review with narrative synthesis using the WHO International Classification of Functioning, Health and Disability

Authors: Victoria Harbottle, Bronia Arnott, Chris Gale, Elizabeth Rowen, Niina Kolehmainen

Victoria Harbottle (Corresponding author)

Great North Children's Hospital, Royal Victoria Infirmary, Queen Victoria Road, Newcastle upon Tyne, NE1 4LP

Population Health Sciences Institute, Newcastle University, Richardson Road, Newcastle University, Newcastle upon Tyne, NE2 4AX, United Kingdom

victoria.harbottle@ncl.ac.uk

Bronia Arnott

Population Health Sciences Institute, Newcastle University, Newcastle upon Tyne, United Kingdom

Chris Gale

Neonatal Medicine, School of Public Health, Faculty of Medicine, Imperial College London, London, United Kingdom

Elizabeth Rowen

Great North Children's Hospital, Newcastle upon Tyne, United Kingdom

Niina Kolehmainen

Population Health Sciences Institute, Newcastle University, Newcastle upon Tyne, United Kingdom

Confidential: For Review Only

Great North Children's Hospital, Newcastle upon Tyne NHS Hospitals Trust, Newcastle upon Tyne, United Kingdom

Word count (excluding title page, abstract, references, figures and tables): 3227

Confidential: For Review Only

ABSTRACT

Background

Indicators of child health have the potential to inform societal conversations, decision making, and prioritisation. Paediatric core outcome sets are an increasingly common way of identifying a minimum set of outcomes for trials within clinical groups. Exploring commonality across existing sets may give insight into universally important and inclusive child health indicators.

Methods

A search of the Core Outcome Measures in Effectiveness Trial register from 2008 to 2022 was carried out. Eligible articles were those reporting on core outcome sets focused on children and young people aged 0-18 years old. The International Classification of Functioning, Disability and Health (ICF) was used as a framework to categorise extracted outcomes. Information about the involvement of children, young people and their families in the development of sets was also extracted.

Results

206 articles were identified, of which 36 were included. 441 unique outcomes were extracted, mapping to 22 outcome clusters present across multiple sets. Medical diagnostic outcomes were the biggest cluster, followed by pain, communication and social interaction, mobility, self-care and school. Children and young people's views were underrepresented

across core outcome sets, with only 36% of reviewed studies including them at any stage of development.

Conclusions

Existing paediatric core outcome sets show overlap in key outcomes, suggesting the potential for generic child health measurement frameworks. It is unclear whether existing sets best reflect health dimensions important to children and young people, and there is a need for better child and young person involvement in health indicator development to address this.

What is already known on this topic?

- There are aspects of children and young people's health that are not routinely, universally measured
- This lack of data about children and young people's health can hinder health policy, care and research
- Core outcome sets are a way of providing guidance on what to measure, but often relate to specific clinical populations only

What this study hopes to add?

- This study identifies common, universally shared themes across core outcome sets
- These themes help identify key universal indicators of child health that should be measured across populations
- The study further identifies gaps and areas for improvement in the involvement of children and their families in identifying key health indicators

Confidential: For Review Only

INTRODUCTION

Measure what is measurable, and make measurable what is not. (Isaac Newton)

How society measures characteristics of health is important because the act of measuring is an act of making an aspect of health visible—in societal discussion, decision making, and resource allocation. Measurable health characteristics are often referred to as indicators, and how they are defined for measurement shapes the information that is collected and available for decisions. Measuring health through routine and other large-scale data sets is increasingly common, and the use of the resulting data in societal conversations, decision making, and prioritisation likely has major consequences for people's lives.

Many important aspects of child health are not yet routinely measured. While some health indicators such as births, deaths and vaccinations are long-established, many others lack robust data. The Covid-19 pandemic provides an illustrative example, where the absence of data about children's physical and mental health, and development has hindered analyses of the impacts of the pandemic on children and, arguably, limited families', decision-makers', and professionals' ability to articulate their concerns. This, in turn, has hindered effective decision-making about important policy decisions such as opening of schools. The need to re-think the measurement of child health is gaining policy traction internationally[1-3] and the choice over what indicators to measure is now paramount.[4]

Measuring child health is hindered by lack of agreement about the important, universally applicable and clinically meaningful indicators of child health and well-being. The past decade has seen a positive, increased focus on common research outcomes relevant across professional disciplines, illustrated for example in the development of core outcome sets. Core outcome sets provide an agreed, standardised minimum set of outcomes to be

Confidential: For Review Only

reported for a specific clinical group in clinical research and increasingly in clinical practice.[5] There is no standardised methodology for core outcome set development, but they generally involve stakeholders agreeing the most important outcomes to report using consensus methodology [6]. However, although patient involvement in core outcome set development has been recognised important,[7] the sets continue to be fundamentally organised around clinical and diagnostic groups to service healthcare and trials. They do not currently provide an off-the-shelf set for universally important indicators of child health that could be applied across diagnostic categories, and across healthy and clinical populations.

We believe there is a need for a generic, universal, inclusive set of core child health and well-being indicators, and we think such a set should: (i) take the child's perspective (as opposed to e.g. medical or educational); (ii) adopt a life-course view, i.e. consider future health and well-being prospects as well as present health; (iii) be practically oriented, with real potential to inform routine data collection and societal discourse, policy, and interventions; and (iv) be inclusive of diverse health and developmental trajectories.

As the first step, in 2017-2020, we engaged with young people, families and decision-makers to discuss broad ideas about health indicators that might matter to them. Informed by those discussions, the present study sought to identify common themes in existing paediatric core outcome sets, as a way to contribute to the wider efforts to progress towards a generic, universal, inclusive set of core child health and well-being indicators. The present paper reports on two specific objectives: (i) to identify common indicators of health included in published paediatric core outcome sets for children and youth (0-18yrs); and (ii) to explore how children, young people and their families' perspectives informed the selection of these outcomes.

METHODS

Confidential: For Review Only

This study used established review methods[8 9] to identify and select papers reporting on published paediatric core outcome sets. Narrative synthesis was used to analyse the data [10]. A protocol was agreed by the review team, with objectives, selection criteria and data extraction techniques agreed in advance. The results are reported in accordance with the Preferred Reporting Items for Systematic Reviews and Meta Analyses (PRISMA-) 2020 statement.[11] The review objectives fell outside the scope of the International Prospective Register of Systematic Reviews (PROSPERO).[12] We used the WHO International Classification of Functioning, Disability and Health (ICF),[13] an existing consensus framework of health, . The ICF is an internationally recognised set of domains of health and functioning that provides a structured way to understand and classify an individual's health and functioning. It considers health indicators relating to body functions and structures, activities and participation and provides multidisciplinary terminology to classify them [14].

Search

Paediatric core outcome sets were identified from the Core Outcome Measures in Effectiveness Trial (COMET) register.[15] The register is the internationally recognised database of ongoing and completed studies relating to core outcome set development. It is updated by systematic review annually, and by authors registering their sets on an ad hoc basis. The last review carried out by COMET, identifying new published core outcome sets for addition to the database was published 01/2021 including studies up to and including 12/2018.[16] To identify paediatric sets, we systematically searched the COMET register[15] from 01/2008 to 03/2022 using the terms indicated in table 1. The search was updated to incorporate the COMET database review up to and including 12/2019.[17]

Selection

Articles were eligible for inclusion in this review if: (i) they reported on a disease or diagnostic-group specific core outcome sets developed using consensus methods (defined as reporting a clear criteria for determining outcome inclusion/exclusion, the number of

https://mc.manuscriptcentral.com/bmjpo

Confidential: For Review Only

people in each stakeholder group at each stage, and all outcome scoring[7]); (ii) focused on children and young people (0-18yrs) as the health beneficiaries; AND (iii) was published after 2008 (a previous systematic review[18] identifies work prior to this date). Systematic reviews, core outcome set protocols, and core outcome sets without reported consensus methods were excluded, as were articles reporting core outcome sets spanning children and adults. Titles and abstracts were first independently reviewed by one author (VH) with a randomly selected 25% double screened by a second author (ER). Full texts of articles meeting the inclusion criteria were then further reviewed, with a randomly selected 50% double screened. Reasons for exclusion were recorded at both stages. Agreement between authors at screening and full text stages was 94% and 82% respectively, and discrepancies were resolved through discussion.

Data extraction and analysis

Study-specific forms were developed, and piloted; and subsequently used by two authors (VH, ER) to independently extract data on: authorship; the core outcome set scope, use, and outcomes listed; and consensus methodology used, including method type, stakeholder numbers, and patient/public involvement. The ICF was used as a framework for coding the outcome data; this provided a common, internationally agreed terminology for naming and describing health outcomes. Outcomes were considered health indicators if they directly related to the child. Outcomes related to care inputs and processes (e.g., resources, length of stay, attendance) were excluded. Outcomes were coded using a published ICF decision tree.[14] For the full mapping, please see the full data set.[19] Article screening was done in EndNote; data extraction in Microsoft Word; and analysis in Microsoft Excel.

RESULTS

We identified 206 records, of which 68 duplicates were removed. A further 86 records were excluded following title and abstract screening, 16 were excluded following full text assessment (Figure 1), and 36 studies were selected for inclusion in full review (Table 2).

Confidential: For Review Only

Selected articles described 36 core outcome sets related to: gastrointestinal conditions (n=8); neurological conditions (n=7); ear, nose, and throat (n=5); orthopaedics (n=4); general paediatrics (n=3); neonatology (n=2); respiratory (n=2); metabolic disease (n=2); and rheumatology, oncology and dentistry specialities (n=1 each) (Table 2). The number of outcomes in a core set ranged from three to 39, with a median of nine.

Common outcome domains

From the 36 core outcome sets included, 441 outcomes were extracted. Mapping these to the ICF resulted in 22 clusters of outcomes, linked to 25 unique ICF codes (Table 3). Medical diagnoses formed the largest cluster, activity and participation forming the majority of the other larger clusters. Several smaller clusters related to body functions were identified as well as a cluster relating to growth. Personal factors relating to emotional wellbeing were clustered and showed relatively higher commonality. Environmental factors were included in several sets and collectively formed one, large cluster. Table 4 summarises the top 10 clusters by size after medical diagnostic outcomes and shows how many sets each outcome cluster was represented in.

Stakeholder involvement

Table 5 summarises the stakeholder involvement in the three main sections of core outcome set development: (i) generation of an outcome longlist; (ii) consensus process; and (iii) final consensus meeting. The diversity of stakeholders involved varied (Table 5). Five (14%, 5/36) only sought opinion from clinicians/researchers throughout their development, including no parents or children or young people (CYP) at any stage. The remaining thirty-one (86%, 31/36) included parents/caregivers in at least one part of the development, with sixteen including parents/caregivers in both outcome listing and consensus stages. Eleven (31%, 11/36) included CYP in at least one part, with nine (25%, 9/36) including them in both outcome listing and consensus (Table 5). Seven sets related to neonatal or infant populations; three of these were for conditions likely to impact into later childhood

https://mc.manuscriptcentral.com/bmjpo

Confidential: For Review Only

(gastroschisis, brachial plexus injury, infants receiving neonatal care) and so could have considered including older CYP. Parent/caregiver and young person stakeholder representation had increased over time (Figure 2).

The median number of rounds used for consensus methodologies was two (range 1-7), with only three studies (8%, 3/36) using only one consensus round. Across the remaining thirty-three studies using multiple rounds, attrition rates of included stakeholders varied.with a mean of 21% (range 0-63%) for clinicians, 29% (range 0-95%) for studies including parents in more than one round and 19% (range 0-73%) for studies including young people in more than one round. Seven (19%, 7/36) mitigated concerns around attrition by recruiting new parent or CYP stakeholder groups for subsequent rounds. Patient and public involvement (PPI) in study design was not universally included; only fourteen (39%, 14/36) included parents or young people, either through inclusion in the study steering group, through consultation with an advisory group or through the piloting of methods. There was some evidence that the use of PPI in designing study methods impacted consensus attrition rates, with mean stakeholder attrition lower for studies that utilised PPI (Figure 3, Table 5).

Figure 4 shows all 36 core outcome sets, plotted by stakeholder (parents and/or children) involvement in longlisting (x-axis) and the consensus process (y-axis). This figure highlights that the sets with greater stakeholder involvement tended to be those including a smaller number of domains. The bubble sizes represent the number of individual outcome domains included in the final core outcome set. Sets nearest the top right-hand corner are those with higher proportions of involvement at both stages of the process (representing higher proportions of stakeholder involvement). Clustered around zero are those sets with very minimal, or no stakeholder involvement at either stage, and those to the left of the plot represent those that used reviews only for the longlisting stage, or for which data around stakeholder participation was unavailable.

Confidential: For Review Only

DISCUSSION

We extracted 441 outcome variables from 36 paediatric core outcome sets, and mapped them to 22 outcome clusters of the International Classification of Functioning, Disability and Health (ICF) as well as on environmental factors potentially affecting these outcomes. These 22 areas represent potential child health indicators for measurement in routine and large-scale data. The most common indicator cluster was a child's diagnosis (in 76% of sets). The second most common was pain (in 33% of sets), followed by activity and participation indicators related to self-care, school, personal well-being, community and civic life (all in 31% of sets), communication and social interactions, and mobility (both in 25% of sets). These were followed by body structure and function indicators relating to sleep (22%), mental functions (16%) and growth (in 11% of sets). In addition, three categories of common indicators residing outside the ICF were identified: mortality (in 33% of sets), dental/oral health (in 8%), and quality of life (in 53% of sets). Overall, while there is an in-principle commitment to patient involvement in core outcome set development, the selection and prioritisation of indicators in the included sets were more informed by clinicians and researchers than children, young people or parents who were under-represented.

The present review used a robust search and data extraction strategy, independent double screening and data extraction by two authors, and a published decision tree for the coding of data on the ICF. The search was limited to the COMET initiative registry, which itself is rigerously updated annually, with the update published as a peer-reviewed systematic review. We can therefore be relatively certain that the present study successfully identified articles up to and including the last update of the regustry (December 2019); the inclusion after that relies on ad hoc author registration and so it is possible that later sets were not included. We did not assess the quality of the paediatric core outcome sets as this was not one of the aims on this review.

Confidential: For Review Only

There were some limitations to this work. The search excluded core outcome sets that spanned both children and adults. This was as the focus was on transdiagnosic health indicators specifc to children and young people. The priorities, life experiences and opinions of CYP differ compared to adults [20] and we felt including studies with a broader age range would have added adult focussed outcomes that were inconsistent with our aims. A further limitation was that whilst independent double screening and data extration was undertaken by two authors, only a randomly selected proportion was reviewed by the second author.

The outcome clusters identified in the present review align with other, concurrent initiatives. To date, two approaches to selecting indicators and outcomes have dominated: the public health, and the clinical. In the public health approach, coarse indicators (e.g. births, deaths, vaccination rates) are selected and used for national and regional reporting and comparison. One such prominent example is the *State of Child Health* by Royal College of Paediatrics and Child Health (RCPCH).[2] This uses key child health indicators to monitor trends and provide policy recommendation across the United Kingdom. Mortality, weight, oral health and mental health are all included, corresponding to some of the domains in the present review. Another example is the Public Health England (PHE) child health indicators, used to monitor trends and inform policy.[21] A challenge with the current public health indicators and related data are children at highest risk of long-term ill health are not well represented – including children with health conditions or marginalised due to sociodemographic circumstances. They are missed from data collection, or the data does not cover key indicators relevant to them, for example, the PHE data does not include pain or sleep. In contrast, in the clinical approach, highly specialised end points (e.g. particular illness symptoms, treatment side effects) are selected on the basis of their relevance to specific interventions and clinical groups; these indicators and measurement rarely touch upon all children universally. There may be a tacit assumption that missing the highest risk children and young people from the public health data is compensated for by the clinical data. However, this is problematic as it neglects some universally important aspects of child health

Confidential: For Review Only

for the clinical populations, and creates a two-strand system where children are viewed through a binary "typical" versus "clinical" lens which is then translated into segregated policy and decision making. For example, the UK Chief Medical Officers developed separate physical activity guidelines for typically developing and disabled children, in large part based on an argument that there was no compatible data to inform a joint, integrated guideline. Similar concerns apply to other major health areas of obesity, smoking and drinking, and uptake of vaccines.

Arguably, both of the public health and clinical approaches have emerged from measuring what can be measured within the current system and with existing instruments and been heavily influenced by the dominant expert paradigms of public health and clinical medicine. Both approaches can been criticised for overlooking important aspects of health and wellbeing valued by CYP, families, and decision makers – and thus for resulting in data and findings with limited ability to inform decision making. In the present review, we sought to adopt a third perspective to complement these two approaches, namely to identify ways forward for making more visible and measurable some of the important aspects of health that are not yet strongly featured in either of the existing approaches. Our findings here suggest that there indeed are shared, recognisable, universal health indicators that are likely to apply both to public health and clinical enquiries, such as self-care, pain, sleep, and social interactions. These findings converge with, and further add to, the recent report from another international group that, independently and concurrently to us, sought to advance the thinking around child health measurement.[22] That concurrent work retained a healthcare paradigm and focused on existing standardised measurement tools that could be used across diagnostic groups (explicitly excluding health indicators that were deemed not yet measurable through standardised instruments). Similarly to our review, they found universally important health indicators such as survival, growth, pain, school attendance, and social functioning. Collectively, the findings from these two studies provide a strong

Confidential: For Review Only

foundation for the development of a universal, common child health indicator framework that spans traditional discipline and sector boundaries to complement existing core outcome sets for interventions as well as inform routine public health data collection. Developing such approach has the potential to facilitate more integrated, inclusive policy, practice and research across child health by focusing attention to universally important health and wellbeing goals that matter to all children, including children who may be clinically unwell.

To progress a meaningful child health indicator framework will require further, substantial development. Key to this will be the involvement of a wider pool of stakeholders, particularly CYP, in deciding what the key indicators should be and how these are best operationalised for measurement and data collection. The findings from the present review align with those of the most recent COMET annual update[17] that found only 16% of paediatric core sets included direct input from CYP. Whilst this is lower than 31% of sets found in this review, the authors included sets spanning both adult and CYP populations, suggesting that with a broader review strategy CYP inclusion is even poorer. CYP and parents often differ in their priorities to health experts,[4 23 24] and it cannot be assumed that parents hold the same views as children and young people.[25] Therefore, including CYP as well as parents is crucial. There is evidence that CYP both understand and are keen to be involved in the development of health indicator and outcome sets[20] and in neonatal or infant conditions involvement of older children with relevant experiences may be useful. A key lesson from the present review is that those engaging higher numbers of CYP differed from standard delphi methodology, perhaps paying particular attention to adapting the methods. Furthermore, focusing on a manageable number of indicators may facilitate stakeholder involvement.

There are three immediately-actionable recommendations from the present study. First, national data set administrators should consider adding pain and sleep as key health indicators. Second, anyone developing core outcome or indicator sets should ensure they

Confidential: For Review Only

involve children, young people and parents – with adaptations to the methods to make this feasible. Third, clinical evaluators should consider inclusion of key, universally important child health outcomes that may be relevant to their interventions but absent from the current core sets.

FUNDING STATEMENT

Victoria Harbottle, HEE/NIHR ICA Predoctoral Fellow (NIHR300363), and Niina Kolehmainen, HEE/NIHR ICA Senior Clinical Lecturer (NIHR ICA-SCL-2015-01-003) were funded by the NIHR for this research project. The views expressed in this publication are those of the author(s) and not necessarily those of the NIHR, NHS or the UK Department of Health and Social Care.

COMPETING INTERESTS

Victoria Harbottle and Niina Kolehmainen were funded by the NIHR for part of this research project. No further competing interests to declare.

CONTRIBUTORS

VH and NK concieved this project. VH developed the review protocol with input from NK and BA. VH and ER undertook title, abstract and full text screening, and data extraction, and VH and NK undertook outcome coding. All co-authors provided input into the mauscript writing.

I, the Submitting Author has the right to grant and does grant on behalf of all authors of the Work (as defined in the below author licence), an exclusive licence and/or a non-exclusive licence for contributions from authors who are: i) UK Crown employees; ii) where BMJ has agreed a CC-BY licence shall apply, and/or iii) in accordance with the terms applicable for US Federal Government officers or employees acting as part of their official duties; on a worldwide, perpetual, irrevocable, royalty-free basis to BMJ Publishing Group Ltd ("BMJ") its

Confidential: For Review Only

licensees and where the relevant Journal is co-owned by BMJ to the co-owners of the Journal, to publish the Work in Archives of Disease in Childhood and any other BMJ products and to exploit all rights, as set out in our licence.

The Submitting Author accepts and understands that any supply made under these terms is made by BMJ to the Submitting Author unless you are acting as an employee on behalf of your employer or a postgraduate student of an affiliated institution which is paying any applicable article publishing charge ("APC") for Open Access articles. Where the Submitting Author wishes to make the Work available on an Open Access basis (and intends to pay the relevant APC), the terms of reuse of such Open Access shall be governed by a Creative Commons licence – details of these licences and which Creative Commons licence will apply to this Work are set out in our licence referred to above.

Confidential: For Review Only

REFERENCES

1. Jacob CM, Baird J, Barker M, et al. The importance of a life course approach to health: chronic disease risk from preconception through adolescence and adulthood. *Geneva: WHO* 2017
2. Royal College of Paediatrics and Child Health. State of Child Health 2020 [Available from: https://stateofchildhealth.rcpch.ac.uk/.
3. United Nations. The Global Strategy for Women's, Children's and Adolescents' Health (2016-2030). 2015
4. Morris C, Janssens A, Shilling V, et al. Meaningful health outcomes for paediatric neurodisability: Stakeholder prioritisation and appropriateness of patient reported outcome measures. *Health Qual Life Outcomes* 2015;13:87. doi: 10.1186/s12955-015-0284-7 [published Online First: 2015/06/26]
5. Meregaglia M, Ciani O, Banks H, et al. A scoping review of core outcome sets and their 'mapping' onto real-world data using prostate cancer as a case study. *BMC Med Res Methodol* 2020;20(1):41. doi: 10.1186/s12874-020-00928-w [published Online First: 2020/02/28]
6. Williamson PR, Altman DG, Blazeby JM, et al. Developing core outcome sets for clinical trials: issues to consider. *Trials* 2012;13(1):132. doi: 10.1186/1745-6215-13-132
7. Kirkham JJ, Davis K, Altman DG, et al. Core Outcome Set-STAndards for Development: The COS-STAD recommendations. *PLoS Med* 2017;14(11):e1002447. doi: 10.1371/journal.pmed.1002447 [published Online First: 2017/11/18]
8. Arksey H, O'Malley L. Scoping studies: towards a methodological framework. *International journal of social research methodology* 2005;8(1):19-32.
9. Peters MDJ, Marnie C, Tricco AC, et al. Updated methodological guidance for the conduct of scoping reviews. *JBI Evidence Synthesis* 2020;18(10) doi: http://dx.doi.org/10.11124/JBIES-20-00167
10. Popay J, Roberts H, Sowden A, et al. Guidance on the conduct of narrative synthesis in systematic reviews. *A product from the ESRC methods programme Version* 2006;1(1):b92.
11. Tricco AC, Lillie E, Zarin W, et al. PRISMA Extension for Scoping Reviews (PRISMA-ScR): Checklist and Explanation. *Ann Intern Med* 2018;169(7):467-73. doi: 10.7326/M18-0850 [published Online First: 2018/09/05]
12. Chien PF, Khan KS, Siassakos D. Registration of systematic reviews: PROSPERO. *BJOG: An International Journal of Obstetrics & Gynaecology* 2012;119(8):903-05. doi: https://doi.org/10.1111/j.1471-0528.2011.03242.x
13. World Health O. International classification of functioning, disability and health : ICF. Geneva: World Health Organization, 2001.
14. Cieza A, Fayed N, Bickenbach J, et al. Refinements of the ICF Linking Rules to strengthen their potential for establishing comparability of health information. *Disability and Rehabilitation* 2019;41(5):574-83. doi: 10.3109/09638288.2016.1145258
15. The COMET Initiative. [Available from: https://www.comet-initiative.org/ accessed January 2020.
16. Gargon E, Gorst SL, Williamson PR. Choosing important health outcomes for comparative effectiveness research: 5th annual update to a systematic review of core outcome sets for research. *PLoS One* 2019;14(12):e0225980. doi: 10.1371/journal.pone.0225980 [published Online First: 2019/12/13]
17. Gargon E, Gorst SL, Matvienko-Sikar K, et al. Choosing important health outcomes for comparative effectiveness research: 6th annual update to a systematic review of core outcome sets for research. *PLOS ONE* 2021;16(1):e0244878. doi: 10.1371/journal.pone.0244878
18. Sinha I, Jones L, Smyth RL, et al. A Systematic Review of Studies That Aim to Determine Which Outcomes to Measure in Clinical Trials in Children. *PLoS Medicine* 2008;5(4):e96. doi: 10.1371/journal.pmed.0050096

Confidential: For Review Only

19. Harbottle V, Kolehmainen N. Paediatric Core Outcome Sets Review Coding. Online data set, 2022.
20. Sherratt FC, Bagley H, Stones SR, et al. Ensuring young voices are heard in core outcome set development: international workshops with 70 children and young people. *Res Involv Engagem* 2020;6:19. doi: 10.1186/s40900-020-00202-9 [published Online First: 2020/05/12]
21. Public Health England. Child Health Profiles 2021 [Available from: https://fingertips.phe.org.uk/profile/child-health-profiles accessed January 2021.
22. Algurén B, Ramirez JP, Salt M, et al. Development of an international standard set of patient-centred outcome measures for overall paediatric health: a consensus process. *Archives of Disease in Childhood* 2021;106(9):868-76. doi: 10.1136/archdischild-2020-320345
23. Singendonk MMJ, Rexwinkel R, Steutel NF, et al. Development of a Core Outcome Set for Infant Gastroesophageal Reflux Disease. *Journal of Pediatric Gastroenterology and Nutrition* 2019;68(5):655-61. doi: 10.1097/mpg.0000000000002245
24. Sinha IP, Gallagher R, Williamson PR, et al. Development of a core outcome set for clinical trials in childhood asthma: a survey of clinicians, parents, and young people. *Trials* 2012;13(1):103. doi: 10.1186/1745-6215-13-103
25. Zeevenhooven J, Rexwinkel R, Van Berge Henegouwen VWA, et al. A Core Outcome Set for Clinical Trials in Pediatric Functional Abdominal Pain Disorders. *The Journal of Pediatrics* 2020;221:115-22.e5. doi: 10.1016/j.jpeds.2020.02.032
26. Allin BSR, Hall NJ, Ross AR, et al. Development of a gastroschisis core outcome set. *Arch Dis Child Fetal Neonatal Ed* 2019;104(1):F76-F82. doi: 10.1136/archdischild-2017-314560 [published Online First: 2018/03/16]
27. Rexwinkel R, Van Berge Henegouwen VWA, Singendonk MMJ, et al. Development of a Core Outcome Set for Children Aged 1-18 Years with Gastroesophageal Reflux Disease. *The Journal of Pediatrics* 2022 doi: 10.1016/j.jpeds.2022.01.043
28. Steutel NF, Benninga MA, Langendam MW, et al. Developing a core outcome set for infant colic for primary, secondary and tertiary care settings: a prospective study. *BMJ Open* 2017;7(5):e015418. doi: 10.1136/bmjopen-2016-015418
29. Karas J, Ashkenazi S, Guarino A, et al. A core outcome set for clinical trials in acute diarrhoea. *Archives of Disease in Childhood* 2015;100(4):359-63. doi: 10.1136/archdischild-2014-307403
30. Kuizenga-Wessel S, Steutel NF, Benninga MA, et al. Development of a core outcome set for clinical trials in childhood constipation: a study using a Delphi technique. *BMJ Paediatr Open* 2017;1(1):e000017. doi: 10.1136/bmjpo-2017-000017 [published Online First: 2018/04/11]
31. Crudgington H, Rogers M, Bray L, et al. Core Health Outcomes in Childhood Epilepsy (CHOICE): Development of a core outcome set using systematic review methods and a Delphi survey consensus. *Epilepsia* 2019;60(5):857-71. doi: 10.1111/epi.14735
32. Joachim KC, Farid-Kapadia M, Butcher NJ, et al. Core outcome set for children with neurological impairment and tube feeding. *Developmental Medicine & Child Neurology* 2020;62(2):201-06. doi: 10.1111/dmcn.14326
33. Murugupillai R, Ranganathan SS, Wanigasinghe J, et al. Development of outcome criteria to measure effectiveness of antiepileptic therapy in children. *Epilepsy & Behavior* 2018;80:56-60. doi: 10.1016/j.yebeh.2017.12.011
34. Nabbout R, Auvin S, Chiron C, et al. Development and content validation of a preliminary core set of patient- and caregiver-relevant outcomes for inclusion in a potential composite endpoint for Dravet Syndrome. *Epilepsy Behav* 2018;78:232-42. doi: 10.1016/j.yebeh.2017.08.029 [published Online First: 2017/11/08]
35. Pease A, Goodenough T, Borwick C, et al. Development of a core outcome set for evaluative research into paediatric cerebral visual impairment (CVI), in the UK and Eire. *BMJ Open* 2021;11(9):e051014. doi: 10.1136/bmjopen-2021-051014

Confidential: For Review Only

36. Butler DP, De la Torre A, Borschel GH, et al. An International Collaborative Standardizing Patient-Centered Outcome Measures in Pediatric Facial Palsy. *JAMA Facial Plast Surg* 2019;21(5):351-58. doi: 10.1001/jamafacial.2019.0224
37. Allori AC, Kelley T, Meara JG, et al. A Standard Set of Outcome Measures for the Comprehensive Appraisal of Cleft Care. *The Cleft Palate-Craniofacial Journal* 2017;54(5):540-54. doi: 10.1597/15-292
38. Balakrishnan K, Bauman N, Chun RH, et al. Standardized Outcome and Reporting Measures in Pediatric Head and Neck Lymphatic Malformations. *Otolaryngology–Head and Neck Surgery* 2015;152(5):948-53. doi: 10.1177/0194599815577602
39. Balakrishnan K, Sidell DR, Bauman NM, et al. Outcome measures for pediatric laryngotracheal reconstruction: International consensus statement. *The Laryngoscope* 2019;129(1):244-55. doi: 10.1002/lary.27445
40. Harman NL, Bruce IA, Kirkham JJ, et al. The Importance of Integration of Stakeholder Views in Core Outcome Set Development: Otitis Media with Effusion in Children with Cleft Palate. *PLOS ONE* 2015;10(6):e0129514. doi: 10.1371/journal.pone.0129514
41. Liu PZ, Ismail-Koch H, Stephenson K, et al. A core outcome set for research on the management of otitis media with effusion in otherwise-healthy children. *International Journal of Pediatric Otorhinolaryngology* 2020;134:110029. doi: 10.1016/j.ijporl.2020.110029
42. Leo DG, Jones H, Murphy R, et al. The outcomes of Perthes' disease. *The Bone & Joint Journal* 2020;102-B(5):611-17. doi: 10.1302/0301-620X.102B5.BJJ-2020-0072
43. Marson BA, Manning JC, James M, et al. Development of the CORE-Kids core set of outcome domains for studies of childhood limb fractures. *Bone Joint J* 2021;103-b(12):1821-30. doi: 10.1302/0301-620x.103b.Bjj-2020-2321.R2 [published Online First: 2021/08/21]
44. de Graaf H, Sukhtankar P, Arch B, et al. Duration of intravenous antibiotic therapy for children with acute osteomyelitis or septic arthritis: a feasibility study. *Health Technol Assess* 2017;21(48):1-164. doi: 10.3310/hta21480 [published Online First: 2017/09/02]
45. Pondaag W, Malessy MJA. Outcome assessment for Brachial Plexus birth injury. Results from the iPluto world-wide consensus survey. *Journal of Orthopaedic Research* 2018;36(9):2533-41. doi: 10.1002/jor.23901
46. Matvienko-Sikar K, Griffin C, Kelly C, et al. A core outcome set for trials of infant-feeding interventions to prevent childhood obesity. *International Journal of Obesity* 2020 doi: 10.1038/s41366-020-0538-2
47. Palermo TM, Walco GA, Paladhi UR, et al. Core outcome set for pediatric chronic pain clinical trials: results from a Delphi poll and consensus meeting. *Pain* 2021;162(10):2539-47. doi: 10.1097/j.pain.0000000000002241
48. Topjian Alexis A, Scholefield Barnaby R, Pinto Neethi P, et al. P-COSCA (Pediatric Core Outcome Set for Cardiac Arrest) in Children: An Advisory Statement From the International Liaison Committee on Resuscitation. *Circulation* 2020;0(0):CIR.0000000000000911. doi: 10.1161/CIR.0000000000000911
49. Bösch F, Zeltner NA, Baumgartner MR, et al. Key patient-reported outcomes in children and adolescents with intoxication-type inborn errors of metabolism: an international Delphi-based consensus. *Orphanet Journal of Rare Diseases* 2022;17(1) doi: 10.1186/s13023-022-02183-2
50. Pugliese M, Tingley K, Chow A, et al. Core Outcome Sets for Medium-Chain Acyl-CoA Dehydrogenase Deficiency and Phenylketonuria. *Pediatrics* 2021;148(2) doi: 10.1542/peds.2020-037747 [published Online First: 2021/07/17]
51. Damhuis SE, Bloomfield FH, Khalil A, et al. A Core Outcome Set and minimum reporting set for intervention studies in growth restriction in the NEwbOrN: the COSNEON study. *Pediatric Research* 2020 doi: 10.1038/s41390-020-01119-5

Confidential: For Review Only

52. Webbe JWH, Duffy JMN, Afonso E, et al. Core outcomes in neonatology: development of a core outcome set for neonatal research. *Archives of Disease in Childhood - Fetal and Neonatal Edition* 2020;105(4):425. doi: 10.1136/archdischild-2019-317501
53. Gilchrist FJ, Ali I, Brodlie M, et al. Developing a core outcome set for children with protracted bacterial bronchitis. *ERJ Open Research* 2020;6(1):00344-2019. doi: 10.1183/23120541.00344-2019
54. Haeusler GM, Phillips RS, Lehrnbecher T, et al. Core outcomes and definitions for pediatric fever and neutropenia research: A consensus statement from an international panel. *Pediatric Blood & Cancer* 2015;62(3):483-89. doi: 10.1002/pbc.25335
55. Heiligenhaus A, Foeldvari I, Edelsten C, et al. Proposed outcome measures for prospective clinical trials in juvenile idiopathic arthritis–associated uveitis: A consensus effort from the multinational interdisciplinary working group for uveitis in childhood. *Arthritis Care & Research* 2012;64(9):1365-72. doi: 10.1002/acr.21674
56. Smaïl-Faugeron V, Fron Chabouis H, Durieux P, et al. Development of a Core Set of Outcomes for Randomized Controlled Trials with Multiple Outcomes – Example of Pulp Treatments of Primary Teeth for Extensive Decay in Children. *PLoS ONE* 2013;8(1):e51908. doi: 10.1371/journal.pone.0051908

Confidential: For Review Only

Confidential: For Review Only

TABLES AND FIGURES

Table 1: COMET Database Search Criteria

Health Area-disease category
Child Health
AND
Publication year
2008 onwards
AND
Published/unpublished
Published
OR
Minimum age
0
AND
Maximum age
18
AND
Publication year
2008 onwards
AND
Published/unpublished
Published

Table 2: Overview of included paediatric core outcome sets

Author/ Year	Scope of set				Size of Set
	Age range	Condition Area	Condition	Use	
Alin et al, 2019 [26]	Birth upwards	Gastrointestinal	Gastroschisis	Research	8
Rexwinkle et al, 2022 [27]	1-18 yrs	Gastrointestinal	Gastroesophageal refulx	Research and clinical guidelines	9
Sherratt et al, 2020	<18 yrs	Gastrointestinal	Uncomplicated appendicitis	Research	14
Singendonk et al, 2019 [23]	‘Infants’	Gastrointestinal	Infant gastroesophageal reflux disease	Research	9
Steutel et al, 2017 [28]	‘Infants’	Gastrointestinal	Infant colic	Research	6
Karas et al, 2015 [29]	‘Paediatric’	Gastrointestinal	Acute diarrhoea	Research	5
Kuizenga-Wessel et al, 2017 [30]	0-18 yrs	Gastrointestinal	Functional constipation	Research	8
Zeevenhooven et al, 2020 [25]	‘Paediatric’	Gastrointestinal	Functional abdominal pain disorders	Research	8

Crudgington et al, 2019 [31]	5-16 yrs	Neurology	Rolandic epilepsy	Research but mentions clinical use (audit, SE)	39
Joachim et al, 2020 [32]	'Children'	Neurology	Neurological impairment and enteral tube feeding	Research	12
Murugupillai et al, 2018 [33]	Two sets: Pre-school <6 yrs School-age 6-18 yrs	Neurology	Effectiveness of anti-epilepsy therapy in children	Research	8
Morris et al, 2015 [4]	'Children'	Neurology	Neurodisability	Informing development of NHS Outcomes Framework	12
Nabbout at el, 2018 [34]	2-18 yrs	Neurology	Dravet Syndrome	Research	5
Pease et al, 2021 [35]	0-18 yrs	Neurology	Paediatric cerebral visual impairment	Research	27
Butler et al, 2019 [36]	0-18 yrs	Neurology	Facial palsy	Quality of care	20
Allori et al, 2017 [37]	'Child'	Ear, nose and throat	Cleft lip/palate	Research + clinical (benchmarkin g)	22
Balakrishnan et al, 2015 [38]	'Paediatric'	Ear, nose and throat	Head and neck lymphatic malformations	Research	31
Balakrishnan et al, 2019 [39]	'Paediatric'	Ear, nose and throat	Laryngotrache al reconstruction	Research and clinical	8
Harman et al, 2015 [40]	<18 yrs	Ear, nose and throat	Otitis media with effusion (in cleft palate)	Research	9
Liu et al, 2020 [41]	<12 yrs	Ear, nose and throat	Otitis media with effusion (in otherwise healthy children)	Research	9
Leo et al, 2020 [42]	'Children'	Orthopaedics	Perthes disease	Research and clinical practice	14
Marson et al, 2021 [43]	5-16 yrs	Orthopaedics	Limb fractures	Research	8 + 1 additional for upper or lower limb
De Graaf et al, 2017 [44]	'Children'	Orthopaedics	Acute osteomyelitis and septic arthritis	Research-specific RCT being planned	11

Confidential: For Review Only

Pondaag and Malessy, 2018 [45]	Not stated	Orthopaedics	Brachial plexus birth injury	Research and clinical- universal dataset	3
Matvienko-Sikar et al, 2020 [46]	<1 yr	General paediatrics	Infant feeding for the prevention of childhood obesity	Research	25
Palermoa et al, 2021 [47]	'Paediatric'	General paediatrics	Chronic pain	Research	4 mandatory, 3 optional
Topjian Alexis et al, 2020 [48]	'Children'	General paediatrics	Cardiac arrest	Research	5
Bösch et al, 2022 [49]	'Paediatric'	Metabolic disease	Intoxication-type inborn errors of metabolism	Research	17
Pugliese et al, 2021 [50]	<12 yrs	Metabolic disease	Medium-chain acyl-coenzyme A dehydrogenase deficiency (MCAD) and phenylketonuria (PKU)	Research	MCAD- 8 PKU- 9
Damhuis et al, 2020 [51]	Birth	Neonatology	Growth restriction in newborns	Research	19
Webbe et al, 2020 [52]	Neonates	Neonatology	Neonatology	Research	12
Gilchrist et al, 2020 [53]	'Children'	Respiratory	Protracted bacterial bronchitis	Research	6
Sinha et al, 2012 [24]	<5 yrs and 5-18 considered separately	Respiratory	Chronic childhood asthma	Research, pilot	4
Haeusler et al, 2015 [54]	'Children'	Oncology	Fever and neutropenia	Research	10
Heiligenhaus et al, 2012 [55]	Implied <16 yrs	Rheumatology	JIA-associated uveitis	Research	7
Smail-faugeron et al, 2013 [56]	Children with primary teeth	Dental	Pulp treatment for primary teeth	Research	5

Confidential: For Review Only

Table 3: Overview of coded and grouped outcomes extracted from paediatric core outcome sets.

Cluster (n total)	ICF code	n	Examples
Communication (n=18)	Voice and speech functions (b3)	7	Articulation, overall speech, speech ability
	Communication (d3)	11	Receptive language skills, listening skills, receptive communication
Self-care (n= 18)	Eating (d550) Drinking (incl. breast feeding) (d560)	9	Breast feeding, child self/assisted feeding, feeding difficulties
	Self-care (d5)	9	Activities of daily living, toileting, safety
Mobility, movement, and related structures (n=19)	Mobility (d4)	10	Gross motor, fine motor, motor impairment, mobility
	Mobility of joint functions (b710)	4	Hip mobility, passive range of movement, active range of movement
	Structures relating to movement (s7)	5	Limb deformity, femoral head shape, arthritic changes to hip
School and learning (n= 19)	School education (d820)	11	School attendance, engagement in school life,
	Learning and applying knowledge (d1)	8	Literacy, academic attainment, school performance
Social (n= 18)	Interpersonal interactions and relationships (d7)	11	Friendships, relationship with parents/siblings, psychosocial development
	Managing own behaviour (d520)	7	Behavioural concerns, behaviour
Community, play and civic life (n= 14)	Community, social and civic life (d9)	14	Sport participation, time away from activities, social activities, play
Growth (n= 12)	Growth maintenance functions (b560)	6	Head circumference, length, adequate growth
	Weight maintenance functions(b530)	6	Weight, weight gain over time, body composition
Pain (n= 17)	Pain (b280-b289)	17	Pain, abdominal pain,
Mental functions	General mental functions (b1)	5	General cognitive ability, cognitive impairment, psychosocial development

Confidential: For Review Only

(n=11)	Specific mental functions (b140-199)	6	Memory, executive function
Sleep (n= 11)	Sleep (b134)	11	Duration of sleep, awakenings from sleep, sleep quality
Function of senses (n= 11)	Hearing functions (b230)	4	Hearing, hearing impairment,
	Seeing functions (b210)	7	Visual acuity, visual performance
Digestive system functions (n= 8)	Digestive functions (b515)	4	Bowel obstruction, vomiting
	Defecation functions (b525)	4	Defecation pattern, stool consistency, defecation frequency
Respiratory functions (n= 4)	Respiration functions (b440)	3	Breathing difficulties, airway compromise, airway obstruction
	Additional respiratory functions (b450)	1	Cough
Skin (n= 2)	Structure of areas of skin (s810)	2	Skin involvement, skin necrosis
Environmental factors (n=33)	e	33	Family life, mother's knowledge of how to offer food, family stress, family quality of life
Medical diagnosis (n=84)	N/A	84	Sepsis, liver disease, infection, seizure
Quality of life (n= 19)	N/A	19	
Death (n= 13)	N/A	13	Death, mortality, all cause mortality, infection related mortality
Ear, nose, throat and dental (n= 12)	N/A	12	Dental health, oral health, occlusion, facial profile, smile, pathologic root resorption
Personal factors relating to emotional wellbeing (n= 17)	N/A	17	Feelings, self-esteem, feelings about having epilepsy, fear of seizures, emotional wellbeing
Other personal factors (n= 10)	N/A	10	Concealment of condition, psychosocial development, attitude towards disease

https://mc.manuscriptcentral.com/bmjpo

Confidential: For Review Only

Table 4: Top 10 outcome clusters by size (excluding medical diagnostic outcomes)

**Cluster**	**Number of outcomes mapped to cluster (n/441)**	**Number of sets represented in (n/n, %)**
Environmental	33	10/36, 28%
School and learning	19	11/38, 31%
Mobility, movement, and related structures	19	9/36, 25%
Quality of life	19	19/36, 53%
Communication	18	9/36, 25%
Self-care	18	11/36, 31%
Social	18	7/36, 19%
Pain	17	12/36, 33%
Personal factors relating to emotional wellbeing	17	11/36, 31%
Community, play and civic life	14	11/36, 31%

Confidential: For Review Only

Table 5: Summary of stakeholder group involvement in paediatric core outcome set development

**Author/year**	**Outcome Listing**	**Stakeholders included (numbers)**				**Consensus method**	**Stakeholders included (numbers participating across all rounds)**				**Final Consensus Meeting**		**Steering Group Explicitly Mentioned**		
		**Clinicians**	**Parents**	**Young People**	**Other**		**Clinicians**	**Parents**	**Young People**	**Other**	**Y/N**	**P/CYP Included?**	**Y/N**	**P/CYP Included?**	**Other Groups**
Alin et al, 2019	Systematic review	-	-	-	-	Delphi	49	22	0	0	▯	Parents only	▯	N	
Allori et al, 2017	Systematic review, qualitative (clinicians only) - interviews, details not published	-	-	-	-	Modified delphi	28	2	1	0	X	-	▯	▯	X
Balakrishnan et al, 2015	Survey	9	0	0	0	Modified delphi	9	0	0	0	X	-	X	-	X
Balakrishnan et al, 2019	Survey	33	0	0	0	Delphi	33	0	0	0	X	-	X	-	X
Bösch et al, 2022	Systematic review, qualitative focus groups	0	26	19	0	Survey (2 rounds)	30	24	9	0	▯	▯	X	-	Survey piloted with stakeholders including CYP
Butler et al, 2019	Review, communicati	18	3	0	0	Modified delphi	18	3	0	0	X	-	▯	X	Patient advisory

Confidential: For Review Only

	on with patient advisory groups														groups consulted- adults with paediatric-onset palsy
Crudgington et al, 2019	Systematic review	-	-	-	-	Delphi	61	16	3		□	□	X	X	Consulted young person's advisory group
Damhuis et al, 2020	Systematic review	-	-	-	-	Delphi	33	14	0	0	□	Parents only	□	□	X
De Graaf et al, 2017	Systematic review	-	-	-	-	Delphi	97	0	0	0	□	Parents + young person representative	X	-	X
Gilchrist et al, 2020	Systematic review, qualitative interviews with parets, clinician survey	20	16	0	0	Delphi	51	0	0	0	□	Parents only	X	-	X
Haeusler et al, 2015	Review of guidelines and consensus statements	-	-	-	-	Delphi	33	3	0	0	X	-	□	X	X
Harman et al, 2015	Systematic review, qualitative interviews	-	43	22	-	Modified delphi	75	35	8	8	□	X	□	□	Study advisory group-invited 1 x CYP but withdrew

Confidential: For Review Only

															Sought advice from diagnosis specific YPAG
Heiligenhaus et al, 2012	Systematic review	-	-	-	-	Delphi, nominal group	16	0	0	0	□	X	X	-	X
Joachim et al, 2020	Systematic review	-	-	-	-	Delphi	15	3	0	0	□	Invited, unable to attend, further remote validatio n with parents	□	X	X
Karas et al, 2015	Systematic review, survey	66	31	0	4	Delphi	64	32	0	0	X	-	□	X	X
Kuizenga-Wessel et al, 2017	Survey	10 9	16 5	50		Modified delphi	50	80	50	0	□	X	X	X	X
Leo et al, 2020	Systematic review, qualitative interviews,	0	18	12	0	Delphi	36	46	18	0	□	Parents only	X	-	X
Lui et al, 2020	Systematic review, qualitative focus groups, examination of tools used in trials currently	0	3	0	0	Survey	81	53	0	0	X	-	X	-	X

Confidential: For Review Only

Marson et al, 2021	Systematic review, qualitative interviews	0	20	20	0	Delphi	11 1	19	0	15	□	□	X	-	Patient, parent and public advisory group consulted
Matvienko-Sikar et al, 2020	Systematic review and stakeholder meeting	12	0	0	0	Delphi	75	2	0	2	□	Parents only	X	-	X
Morris et al, 2015	Systematic review, qualitative focus groups and interviews, delphi-clinicians	19 1	53	54	0	Nominal group	7	5	3	0	X	-	□	□	Pilot to refine young person participation methods
Murugupillai et al, 2018	Survey	32	50	15	0	Delphi	29	42	0	0	X	-	X	-	X
Nabbout at el, 2018	Qualitative semi structured interviews informed by systematic review	4	7	0	0	Modified delphi	7	0	0	0	X	-	X	-	X
Palermoa et al, 2021	Survey	52	90	93	0	Modified delphi	44	85	86	0	□	X	□	X	Patient advisory group consulted
Pease et al, 2021	Review, qualitative interviews	0	18	6	0	Delphi	52	28	0	0	□	Parents only	X	-	2 x patient involvement meetings with

Confidential: For Review Only

															families, parent and CYP involvement in study design
Pondaag and Malessy, 2018	Systematic review then Survey - binary yes/no for inclusion	69	0	0	0	Survey	59	0	0	0	X	-	X	-	X
Pugliese et al, 2021	Systematic review	-	-	-	-	Delphi	16	37	0	0	▯	Parents only	X	-	2 patient partner investigators with lived experience, led family advisory forum
Rexwinkle et al, 2022	Systematic review, health professionals survey and parent/CYP questionnaire	118	146	69	0	Delphi	80	130	77	0	▯	X	X	-	X
Sherratt et al, 2020	Systematic review + qualitative interviews (parents and CYP) (interviews embedded in unpublished					Delphi	110	32	3	0	▯	▯	X	-	Parents and young persons study advisory group

Confidential: For Review Only

	study so no no. available)													
Singendonk et al, 2019	Survey	125	139	0	0	Delphi	89	127	0	0	□	X	X	-	X
Sinha et al, 2012	Survey	46	28	11	0	Delphi - clinicians Survey- parents and CYP	43	50	0	0	X	-	X	-	Pilot phase tested study materials with parents and young people
Smail-faugeron et al, 2013	Systematic review + stakeholder meeting	6	0	0	0	Delphi	52	0	0	0	X	-	X	-	X
Steutel et al, 2017	Systematic review, Survey	133	55	0	0	Delphi	54	43	0	0	□	X	□	X	X
Topjian Alexis et al, 2020	Used adult set, + suggestions from steering committee	18	0	0	0	Delphi	68	6	0	0	X	-	□	X	X
Webbe et al, 2020*	Systematic review x 2 (trials and qual)	-	-	-	-	Delphi	130	53	0	0	□	□	□	□	X
Zeevenhooven et al, 2020	Systematic review + Survey	152	103	50	0	Delphi	104	102	53	0	□	X	X	X	X

Confidential: For Review Only

Table 6: Attrition of different stakeholder groups throughout consensus rounds

Author/year	No. of rounds	Attrition first to last rounds (%)			Notes
		Clinicians	Parents	Young people	
Alin et al, 2019	3	18	48	-	Attempt to recruit young people, unsuccessful
Allori et al, 2017	7	0	0	0	
Balakrishnan et al, 2015	3	33	-	-	
Balakrishnan et al, 2019	2	0	-	-	
Bösch et al, 2022	2	17	11	0	
Butler et al, 2019	7 teleconferences, each followed by a 2 round delphi	Attrition rates not recorded			
Crudgington et al, 2019	2	20	30	0	
Damhuis et al, 2020	3	3	26	-	
De Graaf et al, 2017	2	33	-	-	
Gilchrist et al, 2020	2	22	-	-	
Haeusler et al, 2015	4	15	25	-	
Harman et al, 2015	3	30	-	-	Parents and young people completed 1 round survey only
Heiligenhaus et al, 2012	2 (1 x delphi, 1 x nominal group	12	-	-	
Joachim et al, 2020	1	-	-	-	
Karas et al, 2015	2	9	-	-	Different group of parents invited to round 2
Kuizenga-Wessel et al, 2017	2	-	-	-	Different stakeholder groups of invited to round 2
Leo et al, 2020	2	22	49	33	

Confidential: For Review Only

Lui et al, 2020	1	-	-	-	
Marson et al, 2021	3	31	27	-	
Matvienko-Sikar et al, 2020	3	63	95	0	
Morris et al, 2015	1	-	-	-	1 round nominal group
Murugupillai et al, 2018	2	9	16	-	Young people completed 1 round survey only
Nabbout at el, 2018	2	13	-	-	
Palermoa et al, 2021	2	15	6	8	
Pease et al, 2021	2	35	30	-	
Pondaag and Malessy, 2018	3	14	-	-	
Pugliese et al, 2021	3	48	38	-	
Rexwinkle et al	1	-	-	-	
Sherratt et al, 2020	3	30	44	73	
Singendonk et al, 2019	2	-	-	-	Further clinician recruitment for round 2<br>Different group of parents invited to round 2
Sinha et al, 2012	2	6	-	-	Different group of parents invited to round 2, no young people invited to round 2
Smaïl-Faugeron et al, 2013	3	16	-	-	
Steutel et al, 2017	2	59	-	-	Different group of parents invited to round 2
Topjian Alexis et al, 2020	2	18	0	-	
Webbe et al, 2020	3	13	52	-	Parents and young people combined in one group
Zeevenhooven et al, 2020	2	32	-	-	Different group of parents and young people invited to round 2

Confidential: For Review Only

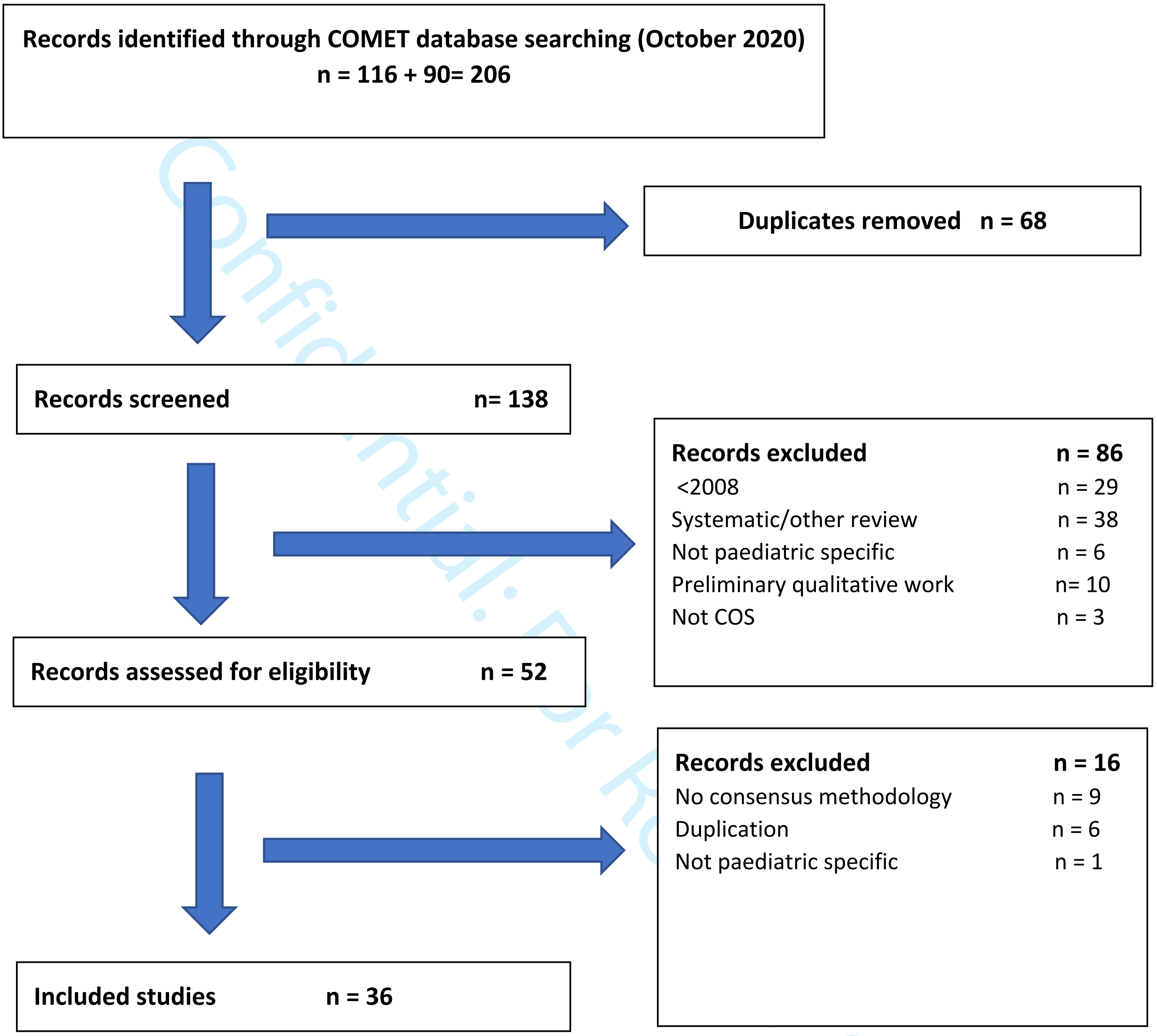

Figure 1: Summary of screening strategy

Confidential: For Review Only

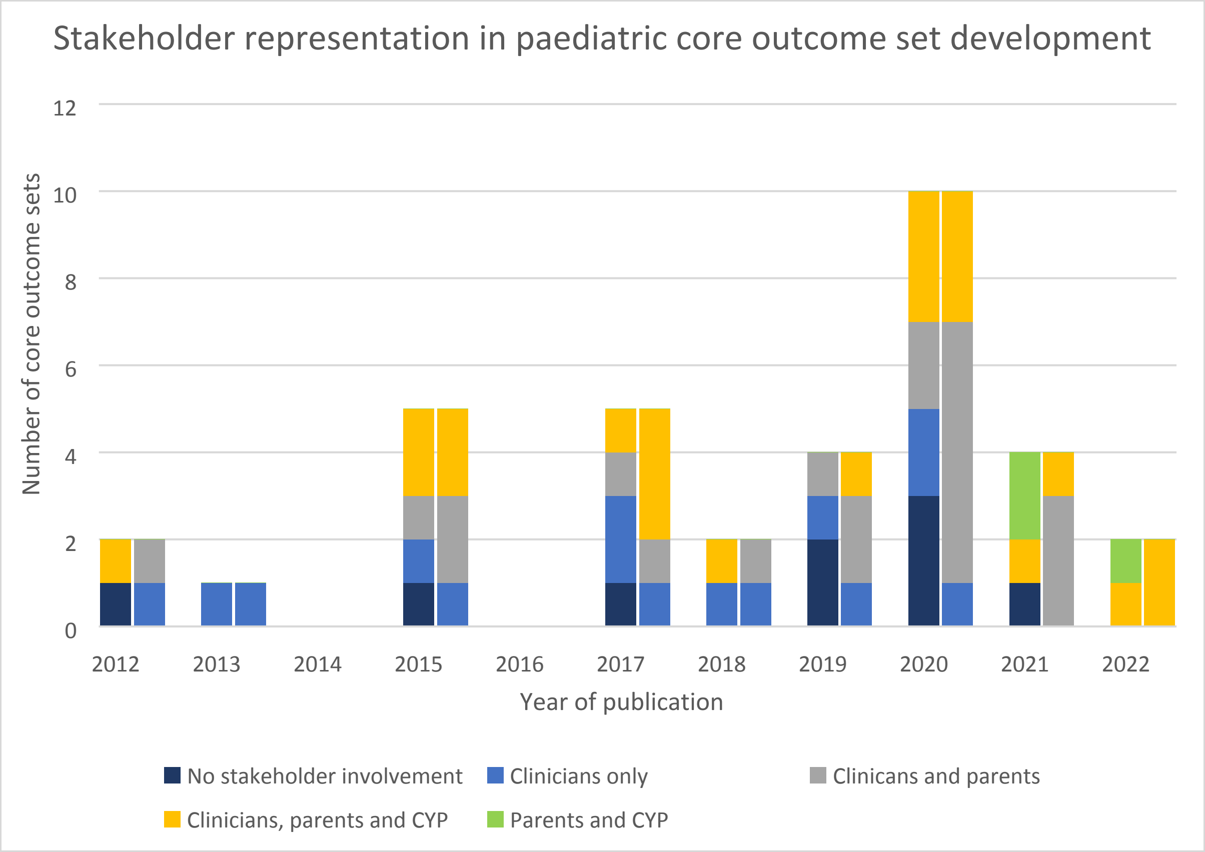

Figure 2: Stakeholder groups involved in (i) outcome long listing and (ii) consensus process by year

Left hand bar represents outcome long listing, right hand bar represents consensus process

For Review Only

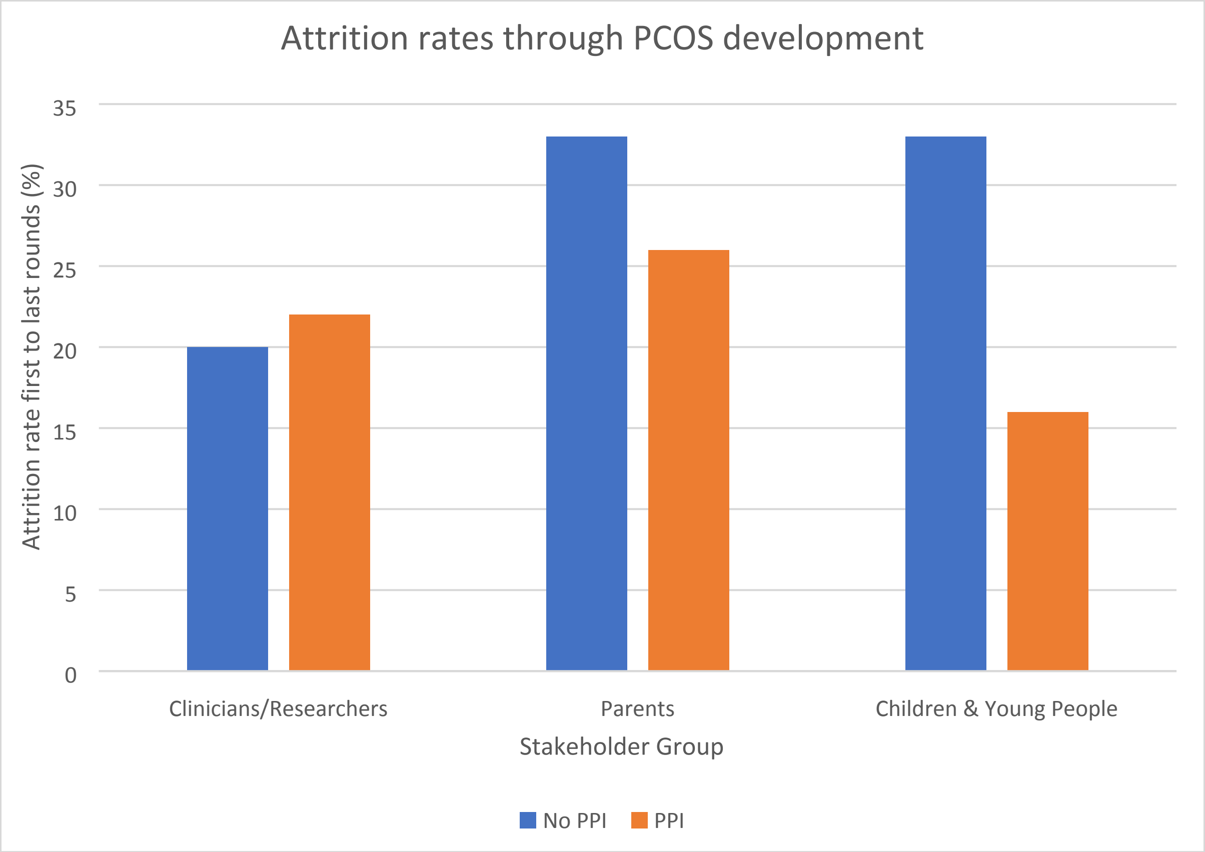

Figure 3: Mean attrition between consensus rounds by stakeholder group, comparing studies including PPI in study design to those without.

For Review Only

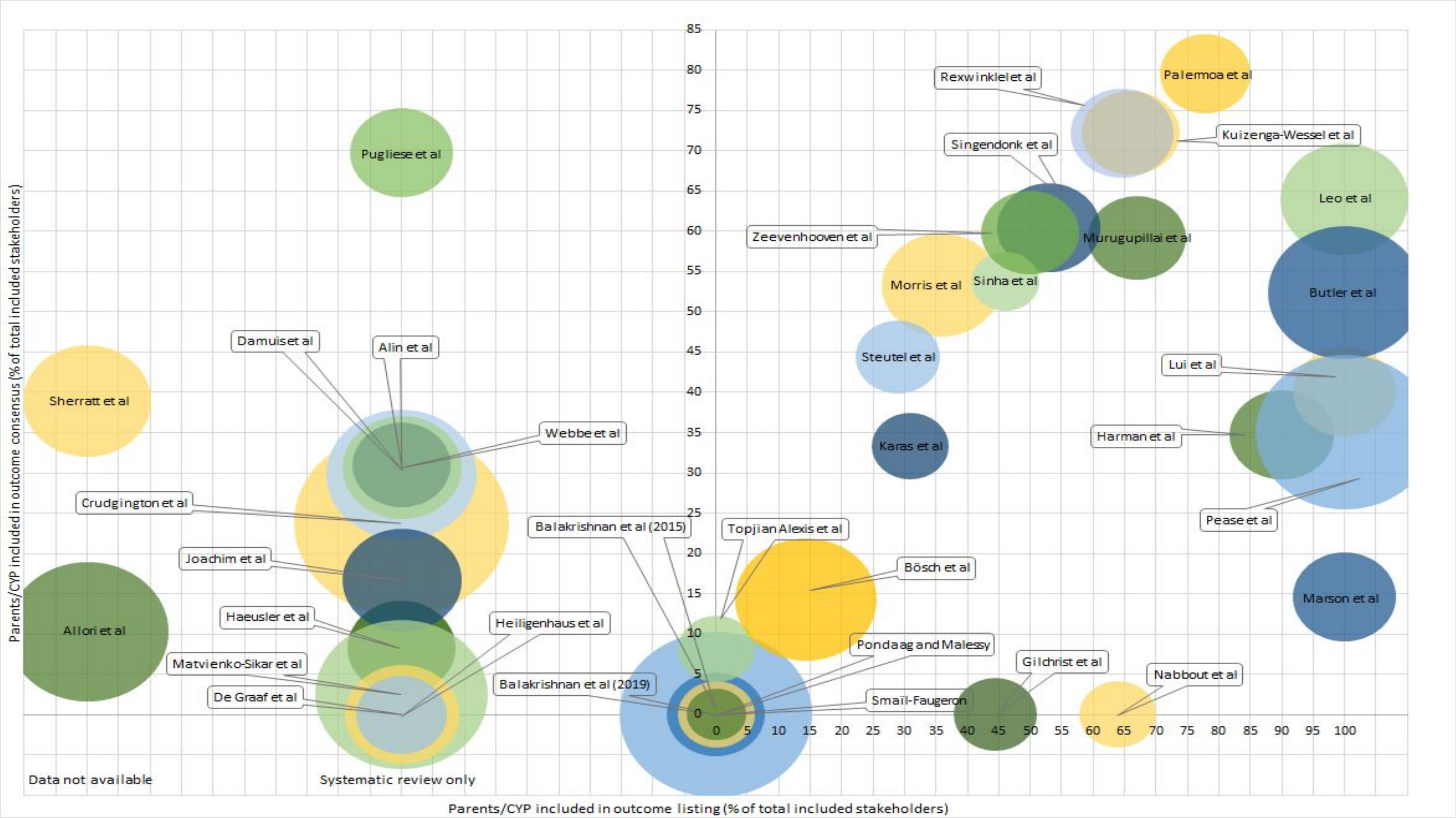

Figure 4: Stakeholder involvement in outcome long listing and outcome consensus for all paediatric core outcome sets. Bubble size represents final paediatric core outcome set size.

Studies to the left of the y-axis had no stakeholder involvement in the outcomes listing phase as outcome longlists were derived from systematic reviews, or the stakeholder breakdown was not published.
